# A Continuum of Evolving De Novo Genes Drives Protein-Coding Novelty in *Drosophila*

**DOI:** 10.1007/s00239-020-09939-z

**Published:** 2020-04-07

**Authors:** Brennen Heames, Jonathan Schmitz, Erich Bornberg-Bauer

**Affiliations:** Institute for Evolution and Biodiversity, 48149 Münster, Germany

**Keywords:** Gene emergence, De novo gene, Orphan gene, Intrinsic disorder, *Drosophila*, Protein evolution

## Abstract

Orphan genes, lacking detectable homologs in outgroup species, typically represent 10–30% of eukaryotic genomes. Efforts to find the source of these young genes indicate that de novo emergence from non-coding DNA may in part explain their prevalence. Here, we investigate the roots of orphan gene emergence in the *Drosophila* genus. Across the annotated proteomes of twelve species, we find 6297 orphan genes within 4953 taxon-specific clusters of orthologs. By inferring the ancestral DNA as non-coding for between 550 and 2467 (8.7–39.2%) of these genes, we describe for the first time how de novo emergence contributes to the abundance of clade-specific *Drosophila* genes. In support of them having functional roles, we show that de novo genes have robust expression and translational support. However, the distinct nucleotide sequences of de novo genes, which have characteristics intermediate between intergenic regions and conserved genes, reflect their recent birth from non-coding DNA. We find that de novo genes encode more disordered proteins than both older genes and intergenic regions. Together, our results suggest that gene emergence from non-coding DNA provides an abundant source of material for the evolution of new proteins. Following gene birth, gradual evolution over large evolutionary timescales moulds sequence properties towards those of conserved genes, resulting in a continuum of properties whose starting points depend on the nucleotide sequences of an initial pool of novel genes.

**Electronic supplementary material:**

The online version of this article (10.1007/s00239-020-09939-z) contains supplementary material, which is available to authorized users.

## Introduction

Taxonomically restricted ‘orphans’, which constitute up to 30% of genes in some eukaryotes (Wissler et al. 2012; Van Oss and Carvunis 2019), are defined by their lack of homologs outside a given phylogenetic distribution. While a definitive explanation for their abundance remains to be found, gene emergence from non-coding DNA may offer at least a partial answer. Now known to occur across eukaryotes, cases of de novo gene emergence have been found in insect, yeast, primate and plant species (McLysaght and Hurst 2016; Schmitz and Bornberg-Bauer 2017; Van Oss and Carvunis 2019). As greater numbers of de novo genes are discovered, questions are raised as to their evolutionary origins, as well as the structural and functional properties of the proteins they encode (McLysaght and Hurst 2016; Schmitz and Bornberg-Bauer 2017; Van Oss and Carvunis 2019).

The first step in identifying de novo-emerged genes is generally to enumerate the set of orphan genes in a given phylogeny. Typically, clusters of orthologous genes (COGs) are identified based on protein sequence homology and defined as taxonomically restricted by lack of homologous proteins in one or more outgroup species. Age is subsequently assigned via phylostratigraphy (Tautz and Domazet-Lošo 2011). However, far fewer studies define the mechanism of origin of each orphan cluster, which requires identification of syntenic DNA in one or more closely related outgroup species (McLysaght and Hurst 2016). While de novo emergence is one explanation for an orphan’s taxonomic restriction, other possibilities include horizontal gene transfer (HGT), N-terminal frameshift mutation, and rapid sequence divergence causing loss of homology signal (Wissler et al. 2013). Distinguishing truly ‘de novo-emerged’ genes from rapidly diverging ones is particularly important because homology, even if hardly recognisable in the face of strong divergence, indicates that an encoded protein has evolved gradually and may have retained structural and functional information (Moyers and Zhang 2017; Casola 2018).

In *Drosophila*, while a number of studies have investigated gene emergence by identifying orphan genes (Chen et al. 2010; Palmieri et al. 2014; Basile et al. 2017), a clade-wide exploration of true de novo gene emergence is still lacking. De novo genes were first discovered in *D. melanogaster* (Begun et al. 2006, 2007; Levine et al. 2006), and while they have since been comprehensively studied in the testis transcriptome (Zhao et al. 2014; Witt et al. 2019), and a handful of orphans confirmed as de novo in *D. melanogaster* (Zhou et al. 2008; Reinhardt et al. 2013; Zhao et al. 2014; Witt et al. 2019), many studies have stopped short of identifying non-coding DNA in an outgroup and therefore only identify orphan genes, even if sometimes termed ‘de novo’. Furthermore, while a number of studies have combined searches of proteomes and genomes in order to increase the sensitivity of their search (Chen et al. 2010; Palmieri et al. 2014; Basile et al. 2019), the de novo genes they identify have been annotated by exclusion of all homology at the genome level, thereby identifying sequences with unknown origin. By extension, conclusions as to the evolutionary dynamics and sequence properties of de novo-emerged genes based on these gene sets should be treated with caution. In this study, we, therefore, aim to (i) systematically investigate the origins of orphan genes across the *Drosophila* clade and confirm cases of genuine de novo emergence from non-coding DNA, (ii) place the properties of this set of de novo genes in the context of existing knowledge of young proteins in *Drosophila* and other species, and (iii) infer the evolutionary trajectories of proteins emerged from non-coding DNA.

While there are a number of outstanding questions to be addressed in the field of de novo gene emergence, thematically they can be divided into two broad categories. The first debate centres on how non-coding regions of the genome acquire protein-coding capacity. This transition requires at a minimum the gain of an open reading frame (ORF), as well as stable transcription, ribosome binding and translation (Cai et al. 2008; Schmitz and Bornberg-Bauer 2017). Transcript-level expression may be a key factor in gene emergence; here, we refer to stable transcription as that which can be distinguished from noise. Orphan genes in *D. melanogaster* have previously been shown to be expressed at a higher level than intergenic regions (Palmieri et al. 2014). Also apparent is the abundance of ‘pervasive’ or ‘spurious’ expression which has been shown to expose entire genomes to expression over short evolutionary timescales (Neme and Tautz 2016). In light of this genome-wide spurious transcriptional activity, it appears that the gain of transcription is unlikely to be a rate-limiting step in the gene birth equation (Neme and Tautz 2016). In addition, the potential of non-coding RNA (ncRNA) to form a functional intermediate may shield a locus from being purged by neutral drift (Ruiz-Orera et al. 2014), making a transcription-first model of gene emergence an attractive hypothesis. A ‘proto-gene’ model of gene birth has also been proposed, where transcription and ORF structure mature in a cooperative process (Carvunis et al. 2012). The most recent evidence indicates that, at least in rice, gain of transcription is frequently the first step in the gene birth process (Zhang et al. 2019). If this is the case, a ‘transcript first’ emergence model may reflect stochastic transcript and ORF turnover, with the higher turnover of transcription making it more likely to occur first.

The second major theme concerns the structural and biophysical properties of the proteins encoded by de novo genes. Central to this are varied findings on the level of structural disorder associated with de novo proteins. While elevated disorder relative to conserved proteins has been found for new domains in *Drosophila* (Bitard-Feildel et al. 2015), evidence in yeast and rodents is discordant. In yeast, *Lachancea* de novo genes were found to be more disordered than conserved genes (Wilson et al. 2017), while *Saccharomyces* de novo genes have been found to have both comparable (Ekman and Elofsson 2010; Vakirlis et al. 2018) and higher disorder relative to conserved genes (Wilson et al. 2017). Similarly, in mouse, orphan and de novo genes have been found to have higher (Wilson et al. 2017) and comparable (Schmitz et al. 2018) levels of disorder, calling into question the claims for strong adaptive causes for trends in disorder and other biophysical properties. One such theory posits that the primary selection on newly born proteins is the avoidance of aggregation (the ‘do no harm’ hypothesis), and that genes may undergo ‘preadaptation’ before fixation, so that only those proteins with the least harmful effect on the cell become fully fledged genes (Ángyán et al. 2012; Wilson et al. 2017). Whether elevated disorder in newly born proteins reflects selection for disorder, against aggregation, or is instead a neutral consequence of the processes of gene birth and fixation remains to be clarified (Nielly-Thibault and Landry 2019). However, the confounding effect of GC-content on protein disorder and aggregation propensity, due to the GC richness of codons for disorder-promoting amino acids, further complicates matters. Basile et al. (2017) have shown that in many taxa, the higher disorder seen in young proteins can be attributed to elevated GC-content, while in rodent species, Casola (2018) concluded that higher disorder was driven by a small number of orphans found to overlap older genes in an alternate reading frame. This view is also supported by recent findings in *Lachancea* yeast that de novo genes emerge preferentially from high GC-content regions, explaining their elevated disorder in comparison to intergenic regions (Vakirlis et al. 2018).

In this study, we systematically investigate the origins of a large number of orphan genes in the *Drosophila* clade and find evidence that up to 39% of the 6297 orphan genes may have originated from ancestrally non-coding regions of the genome. By comparing the annotated proteomes of twelve *Drosophila* species and three outgroup species, and mapping orphan proteins to outgroup genomic sequences, we exploit the short divergence times between species to identify their mechanism of origin. In doing so, we identify a more reliable set of de novo genes than those that have been found to date, given that previous clade-wide studies have identified orphan genes without investigating their roots. We then make use of this set of de novo genes to investigate expression patterns, evolutionary rate, and sequence properties of their encoded proteins, finding evidence that annotated de novo proteins have strong signatures of transcription and abundant translational evidence. Furthermore, we show that their sequence properties appear to fall midway along an evolutionary continuum ranging from the least gene-like sequences to the most conserved sequences—demonstrating for the first time a continuum model of gene emergence in *Drosophila*, as has previously been observed in yeast (Carvunis et al. 2012).

## Results and Discussion

### Orphan Genes form a Significant Fraction of *Drosophila* Genomes

**Fig. 1:**
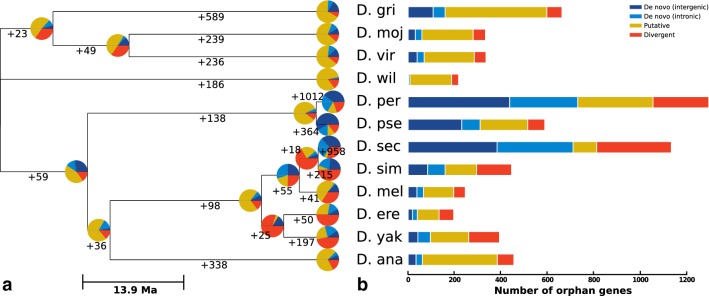
De novo gene emergence is prevalent in the *Drosophila* clade and may explain the abundance of orphan genes. **a** Starting from the annotated proteomes of twelve *Drosophila* species, we identify 6297 orphan genes within 4953 taxon-restricted clusters. Of these orphans, we estimate that up to 39% may have emerged from non-coding DNA. Inferred emergence mechanisms for orphans gained on each branch are illustrated with pie charts. Numbers indicate total orphan gain along each branch. **b** Bars illustrate the total number of orphan genes in each species aggregated along branches

Starting from the annotated proteomes of twelve *Drosophila* species and three outgroup species (Table 1), we clustered sequences by all-vs-all BLASTP, before filtering against the NCBI non-redundant database to remove ancient genes. Using a phylostratigraphic method (Domazet-Loso et al. 2007), we assigned ages to each cluster of orthologs (COG) restricted to the *Drosophila* clade. In this way, a minimum age is assigned parsimoniously, assuming that gene gain occurred along the branch leading to the common ancestor of the species with orthologs in a given COG. Given our aim to identify de novo genes, at this point, we also excluded orphan genes with annotated Pfam domains, which would most likely result from divergence from a conserved (i.e., old) protein. Shown in Table 1 are the total number of protein-coding and orphan genes in the twelve species. Figure 1a illustrates the rates of orphan gain on each branch, along with the proportion of genes gained by each emergence mechanism (see next subsection). The rate of gene gain is seen to be highest on the youngest branches of the tree, pointing to a high rate of gene birth at the time around speciation events, followed by gradual loss of the majority of these genes over the course of millions of years (Tautz and Domazet-Lošo 2011; Schmitz et al. 2018). As seen in Fig. 1b, the total number of orphan genes found in each species is variable, which we hypothesise to be due to a combination of lineage-specific gene loss and variable annotation quality. However, adaptive gene gain may also play a role and could offer a partial explanation for the high number of species-specific orphans found in *D. sechellia*, given the recent adaptation to its toxic host *Morinda citrifolia* (Lavista-Llanos et al. 2014).

**Table 1:** Summary statistics for orphan genes found in the *Drosophila* clade and their inferred mechanism of origination based on sensitive mapping to outgroup genomes using TBLASTN

Species	Proteome	Orphans	De novo (intergenic)	De novo (intronic)	Putative	Divergent	Total de novo (all)	% De novo (all)
*D. ana*	14,365	455	34	27	323	71	61	13.4
*D. yak*	14,824	393	42	54	165	132	96	24.4
*D. ere*	13,605	196	19	21	93	63	40	20.4
*D. mel*	13,907	246	38	28	130	50	66	26.8
*D. sim*	14,179	445	84	75	136	150	159	35.7
*D. sec*	16,465	1133	383	327	102	321	710	62.7
*D. pse*	14,574	588	231	79	205	73	310	52.7
*D. per*	16,874	1294	437	293	324	240	730	56.4
*D. wil*	13,783	217	2	6	179	30	8	3.7
*D. vir*	13,620	335	39	30	216	50	69	20.6
*D. moj*	13,425	333	31	28	221	53	59	17.7
*D. gri*	14,982	662	108	51	438	65	159	24.0
Total	174,603	6297	1448	1019	2532	1298	2467	39.2

### De Novo Emergence Contributes to the Prevalence of Orphan Genes in *Drosophila*

Following identification of orphan genes across the *Drosophila* clade, we investigated their origins by sensitive mapping the proteins from each orphan COG to the twelve *Drosophila* and three outgroup genomes using TBLASTN (Camacho et al. 2009). COGs were annotated as intergenic de novo, intronic de novo, putative de novo, or divergent based on the set of annotated features overlapping with any of the hits in outgroup genomes. We annotated de novo emergence conservatively, with intergenic de novo genes mapping exclusively to gene-free regions in all outgroup species. Intronic de novo genes mapped to at least one intronic region across outgroup mappings, while divergent orphans mapped to one or more exonic features. Strand and frame information was not considered, meaning that some divergent orphans result from out-of-frame or opposite strand exon-overlap (Schmitz et al. 2018). However, a fraction also encompasses rapidly diverging conserved proteins which escape homology detection at the protein level; estimates of the percentage of *Drosophila* genes which may go undetected outside the clade by chance range from 2 to 4%, depending on the *E* value threshold used (Moyers and Zhang 2015). Accordingly, the 1298 (20.6%) orphans we classify as divergent appear to be a reasonable estimate and is also in agreement with synteny-based estimates of ‘divergence beyond recognition’ which suggest a contribution of divergence for up to 20% of orphans (Vakirlis et al. 2020).

In cases where no outgroup hit was found using TBLASTN, COGs were annotated as putative de novo. These unmapped orphan genes may still represent genuine cases of emergence from non-coding DNA, with rapid drift of non-coding DNA in outgroup species explaining the lack of homology signal. However, given their unclear origins, we categorise putative de novo genes separately to avoid including divergent orphans that have lost homology signal at both the nucleotide and protein level. The proportion of orphans gained by de novo emergence along each branch in the *Drosophila* clade is illustrated in Fig. 1a and in total for each species in Fig. 1b. Overall, on the basis of mapping to outgroup non-coding DNA, we find evidence that up to 2467 (39%) of orphans may have emerged de novo, in addition to a comparable number (2532, 40%) of putative de novo genes with unclear evolutionary origin (Table 1).

### Divergence of Outgroup Genomic Regions Limits Inference of the Mechanism of Orphan Gene Emergence

**Fig. 2:**
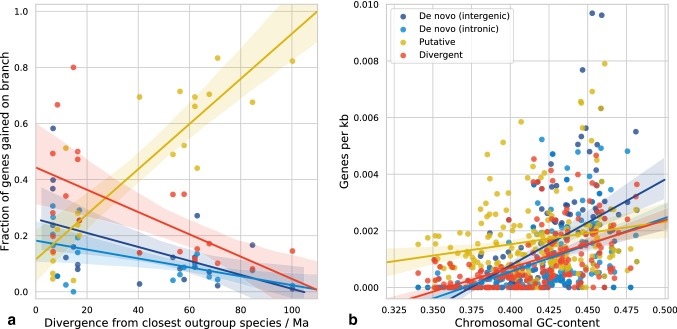
Outgroup genome divergence limits inference of the mechanism of gene emergence. **a** The fraction of orphan genes gained by each mechanism are shown for each branch, with divergence time (*x*-axis) calculated from the root of each branch to the closest leaf; unmapped ‘putative’ de novo genes make up a larger fraction of the most genetically isolated orphans. **b** Orphan gene density correlates with chromosomal GC-content. Orphan gene occurrence is shown for all major *Drosophila* chromosome arms, revealing positive linear correlations with chromosomal GC-content for all classes of gene. The trend for intergenic de novo genes to be found on higher-GC arms (*r* = 0.56, *p* = 3.6e−17) is significantly stronger than that seen for conserved genes (*r* = 0.19, *p* = 0.02; Fig. S1); Fisher r-z transformation *p* = 1.8e−5

To investigate the high number of putative de novo genes found on some branches, we looked at the effects of branch age, branch length, and distance from the root of a branch to its closest extant outgroup species. We find that the total divergence time from the root of a branch to the closest outgroup species to that branch provides the best explanation for differential mapping using TBLASTN. As illustrated by Fig. 2a, the proportion of unmapped orphans shows a strong positive correlation with divergence time (*r* = 0.88, *p* = 1.6e−7). This is reflected in the variable percentage of orphans assigned as de novo by our pipeline in each species (Table 1); *D. willistoni*, with only two de novo genes, is the species with the greatest divergence from any outgroup genome available for mapping (see Fig. 1a). We hypothesise that sequence divergence of syntenic genomic regions in outgroup species underlies this loss of homology signal, possibly driven by the fast divergence of insect genomes (Zdobnov et al. 2002). Additionally, a high rate of outgroup sequence divergence may be best explained by non-coding status in that species, which might point to a majority of putative de novo genes being genuine and therefore not having diverged from an existing gene. Given that a stringent de novo gene identification requires identification of non-coding orthologous DNA (McLysaght and Hurst 2016), our results highlight that the investigation of orphan gene emergence requires a dense phylogeny with as little divergence time as possible between species (Khalturin et al. 2009; Tautz and Domazet-Lošo 2011).

### High Chromosomal GC-Content May Promote Gene Emergence

In light of evidence that GC-rich regions of the genome have higher rates of de novo gene gain (Vakirlis et al. 2018; Wu and Knudson 2018), we next investigated the relationship between mean chromosomal GC-content and the number of orphans per chromosome arm, looking at only the major chromosome arms (> 1000 Mb) in each species. We find a positive correlation between GC-content and the density of orphan genes on a chromosome (*r* = 0.56, *p* = 3.2e−17), as shown in Fig. 2b. The strongest individual relationship is seen for intergenic de novo genes (*r* = 0.56, *p* = 3.6e−17), but weaker positive correlations are seen for other orphan genes (*r* = 0.18–0.51). Since overall gene density is known to correlate with GC-content in mammalian species (Versteeg et al. 2003), we also investigated the relationships between conserved genes and intergenic ORFs with chromosomal GC-content (Fig. S1); in agreement with mammalian species, we find a weak (but non-significant) positive correlation for gene density of genes conserved across *Drosophila* with GC-content (*r* = 0.19, *p* = 0.23). However, the strength of this correlation does not explain the stronger correlation seen for de novo genes (Fisher *r*-*z* transformation *p* = 1.8e−5), which we suggest may stem from a higher rate of gene birth in regions of higher GC-content, in agreement with findings in yeast where de novo gene emergence appears to be promoted near recombination hotspots (Vakirlis et al. 2018; Wu and Knudson 2018). Accordingly, we investigated the role of recombination on *Drosophila* orphans (Fig. S9), finding no significant difference between de novo and conserved genes or intergenic ORFs.

### Lack of ORF Conservation Provides Independent Confirmation of De Novo Gene Emergence

**Fig. 3:**
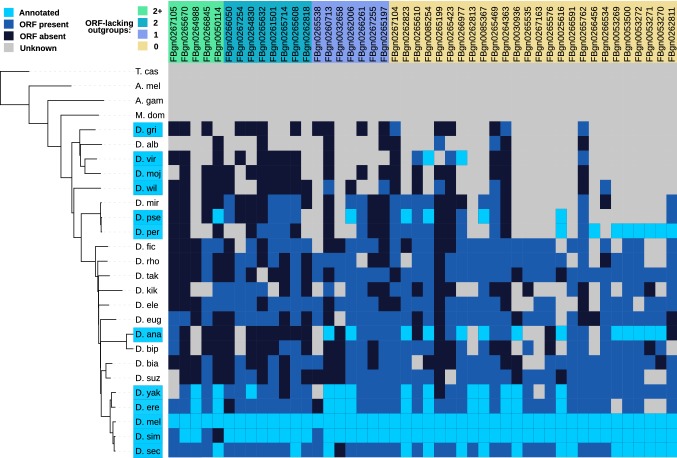
Lack of ORF conservation confirms de novo emergence independently of transcriptional status in outgroups. For single exon de novo genes present in *D. melanogaster*, we extracted syntenic blocks from a 27-way whole genome alignment and searched for ORF presence, scoring species with an ORF overlapping with more than 50% of the *D. melanogaster* ORF as ORF harbouring. Where the alignment was ambiguous (more than 50% gaps), no conclusion as to ORF presence or absence was made (grey). Annotation status in the initial set of twelve *Drosophila* species is shown in light blue. Inference of ORF gain by Dollo parsimony finds 20/46 genes examined to have at least one ORF-lacking outgroup (Color figure online)

To further investigate the role of outgroup divergence on our ability to identify cases of de novo emergence, we examined syntenic regions using a whole genome alignment of 27 insect species (see Materials and Methods). Figure 3 shows the pattern of ORF conservation for single exon de novo genes found in *D. melanogaster*. We find that alignment quality quickly deteriorates as the divergence time to a given species increases, in agreement with the results of TBLASTN mapping. Analysis of ORF conservation also provides further evidence for de novo gene emergence. We used the pattern of ORF presence and absence across the alignment to conservatively infer the point of ORF emergence; where an ORF is present in any of the descendants of a potential non-coding outgroup, the whole group was assigned as coding. We restricted our analysis to single exon de novo genes in order to avoid ambiguity over the splicing of multi-exonic genes in outgroup genomes and additionally only considered genomic regions well aligned to *D. melanogaster* (see Materials and Methods). Summarised in Fig. S2a, we find that of the 46 single exon de novo genes in *D. melanogaster*, 20 lack a syntenic ORF in at least one outgroup branch. We here define ORF presence as alignment of a syntenic ORF with 50% or more of the *D. melanogaster* ORF. Thirteen genes lack an ORF in more than one outgroup, representing the most confident cases of recent ORF formation, while five genes lack aligned syntenic ORFs in more than two outgroups. Importantly, identifying the point of ORF emergence provides additional evidence of de novo gene gain, irrespective of annotation status or expression level (Vakirlis and McLysaght 2019). We note that for genes with only one ORF-lacking outgroup, equal weighting of the likelihood of ORF gain and ORF loss would suggest that just as many ORFs have been lost as gained. Furthermore, while multiple independent losses offer an alternate explanation for the presence of two or more ORF-lacking outgroups, we find ORF gain to be the most parsimonious explanation for our data.

We subsequently extended our analysis of syntenic ORFs by investigating the aligned genomic regions of the 771 single exon de novo genes (from a total of 1063 single exon de novo genes) for which an aligned block of more than one species could be extracted, using a genome alignment of the twelve *Drosophila* species. Taking the same threshold of 50% overlap with the focal ORF to define ORF presence, we are able to identify 172 (22.3%) de novo genes with one or more clear ORF-lacking outgroups, despite the shallower phylogenetic depth of the twelve-way alignment (Fig. S2b). However, given the short length of de novo genes, 50% of the focal ORF length represents a low barrier, and therefore, a stringent test for ORF presence which is likely to capture many short pre-existing ORFs present across the alignment. Using a more relaxed threshold of 80% overlap with the focal ORF, the number of genes with ORF-lacking outgroups increases to 302 (39.2%). Applying the same analysis to intergenic ORFs, we find comparable results, with 580/2439 (23.7%) ORFs having a clear ORF-lacking outgroup using a 50% threshold (Fig. S2b).

We note that identification of an ORF-lacking outgroup is the most stringent test for de novo emergence, since it ignores transcriptional status in outgroup species. Given that our comparative genomics approach to identifying ORF emergence is hampered by the rapid divergence of non-coding syntenic regions in insects (Zdobnov et al. 2002), we chose to keep the full set of 2467 de novo genes classified by TBLASTN mapping, taking into account the non-coding annotation status in outgroup genomes which reflects transcription status in addition to ORF presence or absence. To validate this decision, we later partitioned de novo genes based on ORF synteny (see Fig. S13), finding that the sequence properties of genes with or without an ORF-lacking outgroup do not differ significantly. Additionally, given that the pattern of syntenic ORF presence and absence is comparable for de novo genes and random intergenic ORFs (Fig. S2b), we suggest that the availability of one ORF-lacking outgroup is in this case a reasonable criterion for de novo emergence. Accordingly, we suggest that our figure of 2467 genes is an upper bound for the true number of de novo genes in *Drosophila*. To calculate a conservative estimate for this number, it may be reasonable to extrapolate from the 22.3% of single exon de novo genes found to lack a syntenic ORF; taking this percentage, we arrive at a lower number of 550 de novo genes (0.223 × 2467), or 8.7% or all orphans.

### De Novo Genes Show Robust but Specific Expression

**Fig. 4:**
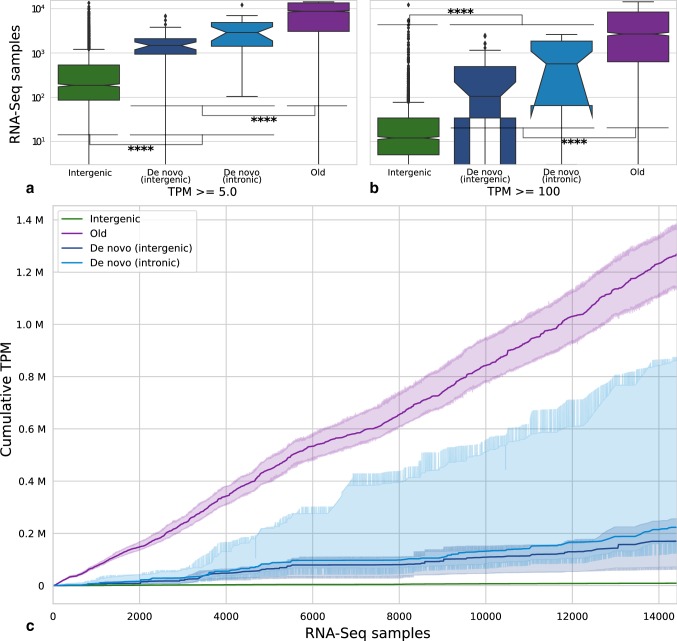
RNA-Seq evidence for de novo genes in *D. melanogaster* across 14,423 RNA-Seq samples. **a** Number of samples in which genes are expressed above a stringent baseline threshold of 5 TPM: 32/66 (48%) de novo genes are found to be expressed in at least 5% of the 14,423 samples. **b** Number of samples in which genes exceed a high expression level of 100 TPM: 34/66 (52%) de novo genes exceed 100 TPM in 10 or more samples. **c** Distribution of cumulative sums of TPM values across all samples: de novo genes are compared to random subsets of 2000 intergenic regions and 2000 old genes. Lines show (median) central tendency for each sequence class within 68% confidence intervals. The total expression of de novo genes across all samples is typically lower than that of conserved genes, but well above background transcription of intergenic regions

We next focused on orphan genes present in *D. melanogaster* to investigate gene expression at the transcript level. In light of the low and generally tissue-specific expression reported for de novo genes (Zhao et al. 2014; Palmieri et al. 2014), we made use of a recent meta-analysis of 14,423 *D. melanogaster* RNA-Seq samples from the Sequence Read Archive (SRA) (Leinonen et al. 2011). The wealth of data across tissues and developmental stages allows us to assess the expression of de novo genes without limitation from low numbers of biological replicate, which may cause transient or weak expression to be missed entirely, as recently demonstrated for orphan genes in yeast (Li et al. 2019). Using transcripts per million (TPM) thresholds of 5 TPM and 100 TPM, for each gene, we calculated the number of samples in which expression exceeded the respective threshold (Fig. 4a, b). We find that, while conserved genes typically exceed both thresholds in many more samples than do the 66 *D. melanogaster* de novo genes, de novo genes exhibit robust expression well separated from intergenic regions. In particular, using a threshold of 5 TPM, we find that 59/66 (89%) de novo genes are expressed in at least 100 samples, and that 32/66 (52%) are expressed at or above this level in at least 711 (5%) of the 14,423 samples. We also note that 5 TPM represents a stringent threshold (Kanitz et al. 2015); taking a more lenient 1 TPM cutoff indicates 54/66 (82%) de novo genes to be expressed in at least 5% of samples. As well as analysing expression on a per-sample basis, we calculated cumulative TPM across all 14,423 RNA-Seq samples for each de novo gene, comparing results to random subsets of 2000 old genes and 2000 intergenic regions. Strikingly, Fig. 4c shows that the median cumulative TPM across all samples for de novo genes is well above the level of intergenic regions, confirming that de novo genes in *D. melanogaster* are unlikely to be flukes of annotation and have the potential to play functional roles. The distribution of final cumulative TPMs for de novo genes illustrates a range of expression levels, with de novo genes generally expressed at a lower level than conserved genes, reflecting their recent birth.

We also analysed a subset of 29 modENCODE mRNA-Seq tissue samples in order to investigate expression strength and tissue specificity. Taking the sum of reads per kilobase per million reads (RPKM) values across these samples confirms that orphan genes show weaker overall expression relative to conserved genes (Fig. S3a, b). As a measure of tissue-specific expression, a Tau score was calculated for each gene, with a value of 1.0 representing expression in only one tissue (Yanai et al. 2005); Figs. S3c and S3d show that orphan genes have more specific expression than conserved genes, in agreement with findings that de novo genes in *D. melanogaster* typically show testis-biased expression (Levine et al. 2006; Zhao et al. 2014; Palmieri et al. 2014). We note that we also identify a higher proportion of de novo genes with testis-biased expression relative to that of the annotated proteome (Mikhaylova et al. 2008), with 8/66 (12%) de novo genes having over 50% of expression biased to testis samples (Fig. S4).

When examining changes in expression with gene age, while no clear trends in expression strength or specificity are for de novo genes over the time span of the *Drosophila* clade (Figs. S3a and S3c), the specificity of older putative orphans is higher than younger, species-specific putative orphans. This is in agreement with the findings of Palmieri et al. (2014) that orphan genes in *Drosophila* with biased expression are more likely to be conserved. As expected, divergent orphans show distributions of strength and specificity values most similar to those of old proteins; given that they overlap with outgroup coding sequence (CDS) regions, many should be found on the same transcripts as those of old genes, explaining their similar expression patterns.

Given the importance of untranslated regions (UTRs) in transcriptional and translational regulation (Moore 2005), we investigated the annotated 5′ and 3′ UTR lengths of orphan genes found in *D. melanogaster* (Fig. S5). We find that the transcripts of de novo genes have shorter 5′ and 3′ UTRs relative to those of old genes (Mann–Whitney *U* for 5′ *p* = 2.4e−7; 3′ *p* = 2.5e−6), while divergent orphans show similar UTR lengths to old genes, in support of their more ancient origins. In summary, the overall weaker expression and less mature transcript structure of de novo genes may be a consequence of their recent evolutionary origin and suggests that expression strength of de novo genes is typically low at the point of gene birth, as suggested by the low expression level of orphan genes in general (Wolf et al. 2009; Carvunis et al. 2012; Palmieri et al. 2014; Li et al. 2019).

### De Novo Genes are Under Weaker Selective Constraint Than Conserved Genes

**Fig. 5:**
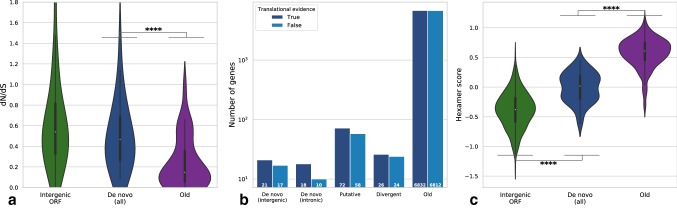
High coding potential for de novo genes found in *D. melanogaster*. **a** The distribution of dN/dS values for de novo genes present in *D. melanogaster* and at least one other species (single exon only) suggests that de novo genes experience lower levels of selective constraint than conserved genes. **b** Aggregated translational evidence for orphan genes in *D. melanogaster*; combining evidence from MS studies and literature sources provides translational support for 39/66 (59%) of de novo genes, compared to ca. 50% of conserved genes. **c** Hexamer scores for de novo genes fall midway between those of random intergenic ORFs and conserved proteins

Orphan genes in *D. melanogaster* have previously been found to be under purifying selection (Palmieri et al. 2014). We therefore searched for signals of selection in the set of de novo genes found in *D. melanogaster* by calculating the ratio of non-synonymous to synonymous codon substitution (dN/dS), from which it is possible to infer selection on protein-coding sequences. We calculated pairwise dN/dS values for all single exon focal ORFs, by aligning them to the least diverged orthologous ORF available (see Materials and Methods). This approach was chosen to allow comparison of de novo and conserved genes to the subset of intergenic ORFs which have an aligned ORF in a sister species (*n* = 4522). Figure 5a shows the distributions of dN/dS value for each sequence class. We find that de novo genes have a marginally lower median dN/dS compared to intergenic ORFs, but both classes appear to be under selective constraint. Interpretation is complicated by the fact that the intergenic ORFs sampled here are also under purifying selection (median dN/dS ca. 0.55). However, at least in *D. melanogaster*, the whole genome has been shown to be subjected to purifying selection (Sella et al. 2009), offering an explanation for the apparent evolutionary constraint on intergenic ORFs. Additionally, a subset of intergenic ORFs may represent emerging de novo genes, in line with a picture of frequent gene emergence from a pool of translated ORFs, as has been evidenced in yeast and mouse (Carvunis et al. 2012; Ruiz-Orera et al. 2018). To help interpret these findings, we carried out an integrative McDonald-Kreitman (iMKT) test for the same sequences, which integrates population-level variation with species divergence to test for adaptive evolution (Murga-Moreno et al. 2019). The iMKT estimate for ɑ, the proportion of non-synonymous sites fixed by positive selection, is intermediate for de novo genes when compared to old genes and intergenic ORFs, but lacks significance (Table S3).

### De Novo Genes in *D. melanogaster* Have Abundant Translational Evidence

We next looked for translational evidence supporting de novo genes as being protein coding, and not just the product of spurious transcriptional activity. We searched three *D. melanogaster* ribosome profiling (Ribo-Seq) datasets for translational support. Due to relatively weak RNA expression, the majority of orphan genes do not appear in these datasets, making it difficult to conclude as to the presence or absence of ribosome binding. However, for those genes present in the RNA-Seq data accompanying the Ribo-Seq datasets (23/66 de novo genes; 359/393 old genes), we searched for evidence of bound and elongating ribosomes. As seen in Fig. S6a, de novo genes have a lower coverage of elongating ribosomes relative to conserved genes, with a distribution of ribosome density similar to that of intergenic ORFs. However, despite having average ribosome density similar to intergenic ORFs, de novo genes are more than twice as likely as intergenic ORFs to have at least one ribosome bound (50.0% vs. 17.9%; Fig. S6b). While ribosome association alone does not confirm functional translation, it may promote translational activity and participate in the gene birth process, as has been demonstrated in yeast (Wilson and Masel 2011). We next searched for mass spectrometry (MS) evidence from two comprehensive *D. melanogaster* proteomics studies (Brunner et al. 2007; Casas-Vila et al. 2017), as well from the SmProt database which includes MS and literature support (Hao et al. 2018) (Fig. S7). We combined evidence from these three sources with the subset of genes found to have non-zero coverage of elongating ribosomes, in total finding 39/66 (59%) of de novo genes to have at least one form of translational support (Fig. 5b; Table S4). Our finding is in agreement with the 36.6% of de novo genes in rice found to have MS evidence by Zhang et al. (2019) and suggests that de novo genes in *D. melanogaster* have strong potential to be translated—although we also note that the level of translational support for the annotated de novo genes studied here may not entirely reflect unannotated de novo genes, of which there are likely many more. To confirm that inclusion of those de novo genes lacking translational evidence or evidence of high transcript expression did not bias our later analysis of sequence properties, we additionally partitioned sequences by high and low expression level (Fig. S12), finding that their properties do not change significantly in either case.

Taking in hand their robust but specific transcription (Fig. 3, S3d), their appearance in Ribo-Seq and proteomics databases (Fig. 5b), and their deviation in hexamer usage from that of intergenic ORFs (next section; Fig. 5c), we infer that many de novo genes are subject to translation and that a proportion may carry out functional cellular roles which remain to be discovered.

### Nucleotide Sequence Properties Reflect the Recent Evolutionary Origin of De Novo Genes

**Fig. 6:**
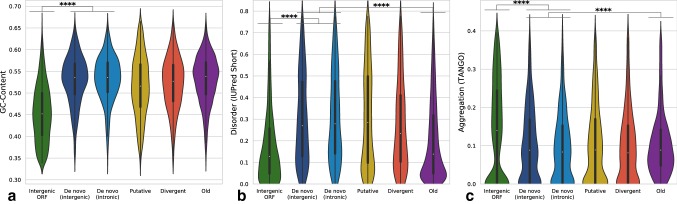
De novo genes in *Drosophila* have higher GC-content than intergenic ORFs and encode more disordered proteins. **a** Intergenic ORFs have markedly lower GC-content than de novo genes (Cohen’s *d* = 1.38, *p* = 1.37e−18), which distribute similarly to conserved genes. **b** Prediction of protein disorder indicates that de novo genes encode more disordered polypeptides than both intergenic ORFs (Cohen’s *d* = 0.63, *p* = 0.0002) and conserved genes (Cohen’s *d* = 0.67, *p* = 1.5e−159). **c** Predicted aggregation propensity reveals that intergenic ORFs encode polypeptides more likely to aggregate than de novo proteins (Cohen’s *d* = 0.64, *p* = 1.4e−308) and conserved proteins

We next analysed the sequence properties of each orphan gene category. As has been shown previously in rice (Zhang et al. 2019), mouse (Schmitz et al. 2018), yeast (Vakirlis et al. 2018) and human (McLysaght and Guerzoni 2015), we find that de novo genes are shorter than conserved proteins, with a median length of 81 residues, but longer than intergenic ORFs (median length 47 residues) (Fig. S10). We subsequently considered nucleotide sequence properties, first analysing hexamer score for the same sequence sets, a measure of similarity of dicodon usage to a set of established genes in a given species (Wang et al. 2013). We find that de novo genes have intermediate hexamer scores relative to old genes and intergenic ORFs, supporting their young age and indicating a gradual process of sequence maturation towards the preferred dicodon usage of *Drosophila* (Fig. 5c). Next, we examined CDS GC-content, finding that all orphan classes show similar levels of GC to conserved genes (Fig. 6a). However, de novo genes show significantly higher GC-content than the set of intergenic ORFs (Cohen’s *d* = 1.38, *p* = 1.4e−18). In light of the higher rate of orphan gain on GC-rich chromosome arms (Fig. 2b), it is possible that biased emergence from regions of higher GC-content may contribute to this trend. Taken together, de novo genes appear to have properties that reflect their young age, being short and more weakly expressed than conserved genes (Figs. S10 and 4), and showing a lower degree of selective constraint (Fig. 5a).

### De Novo Genes Encode More Disordered Proteins Than Both Older Genes and Intergenic ORFs

Having examined the nucleotide sequences of de novo genes, we next predicted the properties of their encoded proteins, examining intrinsic disorder, aggregation propensity, and secondary structure. We find that de novo proteins show elevated disorder when compared to both random intergenic ORFs (Cohen’s *d* = 0.63, *p* = 1.5e−4) and conserved proteins (Cohen’s *d* = 0.67, *p* = 1.5e−159). GC-content is known to have a strong influence on disorder, given that GC-rich codons are also disorder promoting (Ángyán et al. 2012; Basile et al. 2017). However, despite comparable GC-content distributions of de novo and old proteins (Fig. 6a), we see higher disorder in the de novo set (Fig. 6b). We next predicted the aggregation propensity for the same sequence sets. The distribution of aggregation scores is similar for de novo and old proteins (Cohen’s *d* = 0.11, *p* = 5.7e−19), whereas intergenic ORFs show elevated aggregation relative to de novo genes (Cohen’s *d* = 0.64, *p* = 1.4e−308) (Fig. 6c). However, the inherent negative correlation between aggregation and disorder makes it hard to draw conclusions here, and disorder may act as a confounding variable (Ángyán et al. 2012). Lastly, we predicted secondary structure, finding that all gene classes show similar helical and sheet propensity, including random intergenic ORFs (Fig. S11g–i). This is in agreement with findings that secondary structure is already present in random polypeptides (Tretyachenko et al. 2017), and suggests that newly born proteins may represent promising starting points for the evolution of structured and foldable proteins (Bungard et al. 2017).

To control for the effects of nucleotide sequence properties on protein disorder or aggregation propensity, we took subsets of intergenic ORFs and conserved genes matched to the length (Fig. S16) and GC-content (Fig. S17) distributions of the combined set of intergenic and intronic de novo genes (see Materials and Methods). While controlling for length has no major effect on the properties of either sequence class relative to those of de novo genes, when controlling for GC-content, the disorder of intergenic ORFs is seen to increase (Fig. S17d; comparing to Fig. 6b). In other words, the disorder level of intergenic ORFs with the same GC-content as de novo genes is closer to (Cohen’s d for GC-matched sets = 0.15; GC-unmatched sets = 0.63), but still lower than, that of de novo genes. This result is indicative of a disorder-promoting effect of the GC-content of de novo genes, and also suggests that a degree of selection may be acting to further increase disorder, or reduce aggregation propensity, in newly born genes (Ángyán et al. 2012; Basile et al. 2017; Wilson et al. 2017).

## Conclusion

Our results represent the first systematic characterisation of de novo gene emergence in the *Drosophila* clade and confirm previous suggestions that de novo gene emergence is an important underlying cause for the large number of taxonomically restricted orphan genes. Where previous studies have identified a large number of orphans in *Drosophila*, they did not carry out the important additional step of identifying non-coding DNA in one or more outgroup genomes, which provides the best evidence for de novo gain. Here, from over six thousand orphans found across twelve species of fly, we find evidence that de novo birth may explain up to 39% of these genes (Table 1). For the remaining orphans, we categorise the majority as putative de novo genes on the basis of their unknown emergence mechanism. In the absence of identifiable non-coding DNA in an outgroup species, it remains possible that these sequences are rapidly evolving homologs which escape detection at both the protein and nucleotide level. However, we cannot rule out the alternative that many of these genes may too have emerged from non-coding DNA, especially in light of the similarities seen between the putative and de novo genes in terms of length, expression and sequence properties (Figs. S3, S10, S11). However, we here consider putative de novo genes separately to avoid drawing false conclusions regarding the properties of true de novo genes.

Examination of syntenic genomic regions for de novo genes across the clade shows that for up to 40% of de novo genes we can identify at least one outgroup that lacks a syntenic ORF, depending on the alignment depth available (Fig. 3, S2). For these genes, pinpointing ORF formation supports a scenario of de novo emergence without reliance on annotation quality, or knowledge of the expression status in outgroup species (Vakirlis and McLysaght 2019). For the remaining genes, we are unable to unambiguously identify ORF emergence and are therefore reliant on annotation to infer non-coding status in outgroup species. Our figure of 2467, therefore, represents an upper bound for the number of genes classed as de novo. As a lower bound, extrapolating from the 22.3% of single exon genes found to lack a syntenic ORF, we suggest a conservative figure of 550 de novo genes (8.7% of all orphans). However, given our sensitive mapping to outgroup genomes, stringent requirements for orphan classification, and the robustness of gene properties to partitioning based on ORF emergence (Fig. S13), we here classify our set of 2467 mapped de novo genes as likely having emerged from non-coding DNA. While for a subset of these novel genes, we can infer the point of ORF formation, for others it remains ambiguous, likely due to rapid divergence of non-coding syntenic regions—but also suggesting that precursor ORFs may act as a starting point for gradual gene maturation from proto-gene to established gene (Carvunis et al. 2012).

Having identified a clade-wide set of up to 2467 de novo genes, we tried to answer open questions regarding the biophysical characteristics of their encoded proteins, and how they change over evolutionary time. Comparing the sequence properties of de novo genes to those of a set of unannotated intergenic ORFs, we are able to test the null hypothesis that de novo genes remain unchanged from a set of neutrally fixed ORFs. As has been consistently seen before, we find de novo genes to be shorter than older genes. In *Drosophila*, we find that de novo genes do encode proteins with elevated disorder relative to conserved proteins. However, given the high GC-content of de novo genes, elevated disorder is to be expected due to the link between GC-rich codons and disordered amino acids (Basile et al. 2017). In agreement, we find that the higher GC-content of de novo genes relative to intergenic ORFs does appear to promote disorder, but does not alone provide a complete explanation. We, therefore, suggest that selection may act to further increase disorder (or reduce aggregation) at the time of gene birth, beyond that expected for random sequences of a given GC-content (Ángyán et al. 2012; Wilson et al. 2017). Aside from uncertainty over the relationship between GC-content, disorder and aggregation propensity, we find many sequence properties of de novo genes to be intermediate to those of intergenic ORFs and conserved genes. In particular, the distributions of sequence length, hexamer usage and expression level are indicative of the random-sequence origins of de novo genes and lead us to support a model of gradual evolution from an initial pool of novel genes, as has been previously proposed in yeast (Carvunis et al. 2012). We suggest that this reservoir of emerging genes may provide an important source of new proteins in *Drosophila*, a fraction of which gain function and with it the evolutionary stability necessary to avoid loss by genetic drift.

## Materials and Methods

Scripts and data from this study are available online at: https://zivgitlab.uni-muenster.de/ag-ebb/de-novo/droso_de_novo.git

### Orphan Gene Annotation

Genomes, proteomes, CDSs and annotations for twelve species of *Drosophila* (*D. grimshawi*, *D. mojavensis*, *D. virilis*, *D. willistoni*, *D. persimilis*, *D. pseudoobscura*, *D. sechellia*, *D. simulans*, *D. melanogaster*, *D. erecta* and *D. yakuba*) were acquired from FlyBase (r2016_03) (Thurmond et al. 2019). Equivalent data for the three outgroup species (*Anopheles gambiae*, *Lucilia cuprina* and *Ceratitis capitata*) were downloaded from Ensembl Metazoa and the I5K project. For full details of input data and accessions see Table S1. Clusters of orthologous proteins were identified by all-vs-all BLASTP (*E* value cutoff 1e−5) (Altschul et al. 1990). Phylostratigraphy was then performed, assigning gene age based on the phylogenetic distribution of each ortholog cluster (Domazet-Loso et al. 2007). Divergence times for the input species were taken from timetree.org (Hedges et al. 2006). Clusters with an age greater than 50 Mya were discarded, leaving COGs restricted to only *Drosophila* species and not present in outgroups. Remaining clusters were searched by DIAMOND (Buchfink et al. 2015) (*E* value cutoff 1e−3) against the NCBI non-redundant database (Wheeler et al. 2003) to filter out those with ancient homologs. Finally, the Pfam database (Bernsel et al. 2008) was queried to remove any clusters containing proteins with annotated domains, which were considered highly unlikely to have evolved de novo. A list of 50 *Drosophila*-specific Pfam domains were whitelisted (see additional methods accompanying scripts online).

### Outgroup Genome Mapping

The mechanism of origin for each orphan cluster was assigned by identifying non-coding homologous genomic regions in outgroup species to a given cluster. TBLASTN (Camacho et al. 2009) was used to map all protein sequences from each orphan cluster to the genomes of all study species. Where a protein was successfully mapped to an outgroup genome, outgroup status was conservatively annotated by selecting the highest ranked feature intersecting with the mapped coordinates of the protein, on either strand. Default setting for TBLASTN with a protein query against a nucleotide database were used, with a *E* value threshold of 1e-3. Hits across all genomes were subsequently filtered to include only alignments of 20 amino acids or longer. We subsequently analysed all hits remaining in species outside a given orphan COG, assigning an emergence mechanism for each COG in the most conservative way; mapping of any hit from any of the cluster’s sequences to a region annotated as exonic in an outgroup species assigned the cluster as having diverged from an ancestral protein-coding sequence (‘divergent’ orphans). Alternately, if one or more members of the cluster mapped to an intergenic or intronic region of an outgroup genome, it was annotated as intergenic de novo or intronic de novo, respectively. If no homologous outgroup DNA was identified, the cluster was labelled as ‘putative de novo’.

### Preparation of Conserved and Random Control Sequence Sets

We used ORFfinder (Wheeler et al. 2003) to extract all ORFs of 30 nt and longer (with canonical start and stop codons) from all twelve *Drosophila* genomes (> 12 M ORFs). From this set, 12,000 ORFs were picked at random to form a control group. After filtering for ORFs with a whole number of codons, and for those annotated as intergenic in the focal species as well as in all aligned regions across a whole genome alignment of the *Drosophila* clade, we were left with a set of 6763 intergenic ORFs. In a similar fashion, a representative set of old (i.e., conserved) protein sequences was selected from the set of twelve *Drosophila* proteomes by random selection of 6851 proteins, excluding those already annotated as orphans in this study.

### Analysis of Syntenic Genomic Regions

To examine ORF conservation for de novo genes found in *D. melanogaster*, we first used the BioPython AlignIO module (Cock et al. 2009) to extract syntenic alignments from the UCSC 27-way insect whole genome alignment (Rosenbloom et al. 2015). For each locus, the focal species’ CDS coordinates were extended by 2 Kbp (up- and downstream) and were used to extract a multiple sequence alignment of these syntenic regions. To avoid unreliable splicing of outgroup genomes in silico based on the splice sites of the focal gene, only single exon focal genes were considered. We then searched for all ORFs across the alignment. ORFs in the correct orientation and having nucleotide overlap with the focal ORF were kept for further analysis. To account for unreliable alignment, regions with more than half of the alignment gapped relative to *D. melanogaster* were ignored. Species with an overlapping ORF longer than 50% of the *D. melanogaster* ORF were denoted as ORF harbouring. To generate a set of syntenic ORFs for evolutionary rate analysis, the same methodology was applied to a twelve-way whole genome alignment of the *Drosophila* clade (König et al. 2016). In this case, the syntenic regions corresponding to all *Drosophila* single exon orphan genes, old genes and intergenic ORFs in the study set were extracted and syntenic ORFs were identified as before. Syntenic alignments from both the 27-way and twelve-way alignments were subsequently used to calculate pairwise dN/dS values and to identify ORF-lacking outgroups to provide additional evidence for de novo gene emergence. To infer the number of non-coding (ORF-lacking) outgroups, we applied a conservative parsimony approach as has been used before (Zhang et al. 2019); we first mapped unambiguous ORF presence and absence to the *Drosophila* phylogeny, before tracing back from the focal species to identify potential outgroup branches which could be assigned as ORF lacking. Where one or more descendant species in a given outgroup branch was ORF harbouring, the whole branch was conservatively assumed to be ORF harbouring.

### Transcriptional Evidence

Initial *D. melanogaster* expression data were downloaded from with precomputed RPKM values per gene from FlyBase, extracted from the modENCODE tissues project data (SRA accession SRP003905) which include RNA-Seq across 29 tissue samples at a number of life stages. Expression strength was calculated as the sum of RPKM values across samples, while expression specificity was estimated by calculating a Tau score, with a score of 1.0 indicating expression in only a single sample (Yanai et al. 2005). To gain a broader view of expression level across multiple biological replicates, we subsequently made use of a meta-analysis of 14,423 *D. melanogaster* RNA-Seq samples from the SRA database (Leinonen et al. 2011), available for download on the Gene Expression Omnibus (GEO) (accession GSE117217) (Barrett et al. 2013). Raw read counts were converted to transcripts per million (TPM) values to allow comparability across samples, and mean and maximum TPM values were calculate for each *D. melanogaster* gene across all 14,423 samples. Additionally, we calculated cumulative sums of TPM value for each gene across all samples, distributions of which are visualised with median central tendency and 68% confidence intervals computed from 500 bootstrap samples.

### Translational Evidence

Ribosome profiling data from the three available *D. melanogaster* datasets (Dunn et al. 2013; Kronja et al. 2014; Aspden et al. 2014) were downloaded from the GWIPS-viz browser (Michel et al. 2014). In order to find read intersection with the current dm6 *D. melanogaster* gene coordinates, binary files were converted from bigWig to wig format using bigWigtoWig (https://www.encodeproject.org/software/bigwigtowig/), before conversion to BED format using bedtools (Quinlan and Hall 2010). Finally, BED coordinates were remapped from dm3 to dm6 using UCSC’s liftOver executable (Rosenbloom et al. 2015). Read counts for bound and elongating ribosomes were then calculated using the HTSeq python module (Anders et al. 2015), and normalised by CDS length to give values of read count per kilobase. In addition to analysing Ribo-Seq data, we searched for evidence of translation in three additional sources: proteomics evidence was taken from two recent *D. melanogaster* MS studies focused on the whole and developmental proteomes, respectively (Brunner et al. 2007; Casas-Vila et al. 2017), and the SmProt database (Hao et al. 2018), which collates translational evidence for proteins shorter than 100 residues, was parsed for additional MS, Ribo-Seq and literature evidence. These three sources were pooled to identify the full set of *D. melanogaster* protein-coding genes with proteomics evidence, resulting in a list of 6833 unique FlyBase gene identifiers, against which we compared our sets of *D. melanogaster* orphan and conserved genes.

### Evolutionary Rate Analysis

Selective pressure was analysed using PAML’s codeml package (Yang 1997), using the yn00 model (Yang and Nielsen 2000). The analysis was carried out only on *D. melanogaster* single exon orphans, to allow comparison to both older genes as well as intergenic ORFs. Evolutionary rate was calculated in a pairwise manner for each ORF, by alignment of the focal CDS with the least diverged ORF available. The MEGA implementation of MUSCLE (Edgar 2004; Tamura et al. 2007) was then used to generate codon alignments. dN, dS and dN/dS were subsequently calculated for each pairwise codon alignment using the BioPython codeml module (Cock et al. 2009). To handle high values of dN/dS calculated in the case of very low dS, values above 99.8 were discarded. Additionally, we examined population-level selection for *D. melanogaster* genes using the integrative McDonald-Kreitman test (iMKT) (Murga-Moreno et al. 2019). Coordinates for single exon de novo genes, as well as a sample of conserved genes and intergenic ORFs, were remapped to dm5 gene coordinates using FlyBase’s coordinate converter. Variation data were downloaded from PopFly (https://popfly.uab.cat) (Hervas et al. 2017) for the Equatorial Africa metapopulation (EQA), and fasta alignments generated using the supplied script (https://github.com/jmurga/iMKTData/blob/master/src/subsetMultiFasta.py) using the FlyBase r5.57 *D. melanogaster* genome as a reference and *D. simulans* as an outgroup. The iMKT server (https://imkt.uab.cat) was subsequently used to carry out an extended MKT to assess adaptive evolution as well as the fractions of neutral, weakly and strongly deleterious mutations.

### Sequence Property Analysis

Unless otherwise stated, tools were run using default settings. Comparison between sequence sets was made after selection of one protein isoform per gene, and one gene per ortholog cluster. Isoforms were chosen randomly so as to not bias the distribution of sequence lengths, and a representative sequence was taken for each COG by picking the *D. melanogaster* ortholog if present, or else at random. As such, sequence distributions represent distinct evolutionary gains and are not biased by duplication via speciation. Lengths were calculated using an in-house script. GC-content was calculated using the EMBOSS program geecee (Rice et al. 2000). Protein disorder was predicted using IUPred2A (short algorithm) (Mészáros et al. 2018); the number of residues with a disorder score above the recommended threshold of 0.5 was divided by sequence length, to give a proportional disorder score for each protein. Aggregation propensity was calculated using TANGO version 2.3.1 (Fernandez-Escamilla et al. 2004); the number of aggregating residues (with a score above 5%) in stretches of five or more consecutive amino acids was summed and divided by sequence length to give a proportional aggregation score. To calculate hexamer scores, CPAT version 1.2.2 was used with the supplied logistic model for fruit fly (Wang et al. 2013). Protein secondary structure was predicted using the SPIDER3 package (Heffernan et al. 2017). The script ‘SPIDER3-Single_np’ was used without homology assistance, to ensure comparability between sets with varying availability of homologous protein sequences. Repeat content was calculated at the amino acid level using the SEG algorithm (packaged with SLIDER (Peng et al. 2014)). For all sequence properties, outliers more than two standard deviations from the mean were removed. To generate length and GC-content matched subsets of sequences, bins were generated at intervals of 10 amino acids or 5% GC-content, respectively. For each de novo gene, one intergenic ORF and one conserved gene (in the same bin) were selected at random to generate subsets with matching distributions of each sequence property.

### Recombination Rate

Recombination rates for all *D. melanogaster* orphans was calculated using the Recombination Rate Calculator (RRC) (Fiston-Lavier et al. 2010), utilising the experimentally determined crossover rates published by Comeron et al. (2012). Gene start and end coordinates were taken for each *D. melanogaster* orphan, as well as the subset of *D. melanogaster* intergenic ORFs and conserved genes, converted to dm5 coordinates using the FlyBase Coordinate Converter (flybase.org/cgi-bin/coord_converter.pl), and the midpoint recombination rate was found using the RRC server.

### Statistical Tests

All statistical testing was carried out in python. Strength of linear correlations are reported as Pearson’s *r* throughout. To assess the significance of the difference between independent *r* values, a Fisher *r*-*z* transformation was used (available at https://vassarstats.net/rdiff.html). To test for similarity between distributions of various sequence properties, a Mann–Whitney *U* test was carried out, with the likelihood that randomly selected values from one population will differ from a second population reported as a *p* value. Given the large number of data points in many categories, we also calculated effect sizes as an independent measure of similarity between distributions; Cohen’s *d* is reported, ranging from 0 to 2, with 0 indicating no difference in distributions and 2 indicating the most extreme difference. Asterisks are used to illustrate *p* values: **** indicates < 0.0001; *** indicates 0.0001 to 0.001; ** indicates 0.001 to 0.01; and * indicates 0.01 to 0.05.

## Electronic supplementary material

Below is the link to the electronic supplementary material.

Supplementary file1 (PDF 14336 kb)
