# Supplementary material for: A Continuum of Evolving De Novo Genes Drives Protein-Coding Novelty in Drosophila
Source: J Mol Evol. 2020 Apr 7;88(4):382–98. doi: 10.1007/s00239-020-09939-z (PMC7162840; doi:10.1007/s00239-020-09939-z)
Supplement: Supplementary file 1 — Supplementary file1 (PDF 14336 kb) [file 239_2020_9939_MOESM1_ESM.pdf]

Supplementary Figures

Electronic Supplementary Material for the manuscript 'A continuum of evolving de novo genes drives protein-coding novelty in *Drosophila*'

Corresponding author: Erich Bornberg-Bauer (ebb.admin@wwu.de)
Institute for Evolution and Biodiversity, 48149 Muenster, Germany

**Species**	**Release**	**Abbreviation**
D. grimshawi	FlyBase r1.3	*D. gri*
D. mojavensis	FlyBase r1.4	*D. moj*
D. virilis	FlyBase r1.06	*D. vir*
D. willistoni	FlyBase r1.05	*D. wil*
D. persimilis	FlyBase r1.3	*D. per*
D. pseudoobscura	FlyBase r3.04	*D. pse*
D. sechellia	FlyBase r1.3	*D. sec*
D. simulans	FlyBase r2.02	*D. sim*
D. melanogaster	FlyBase r6.11	*D. mel*
D. erecta	FlyBase r1.05	*D. ere*
D. yakuba	FlyBase r1.05	*D. yak*
**Anopheles gambiae*	GCA_000005575.1	*A. gam*
**Lucilia cuprina*	GCA_001187945.1	*L. cup*
**Ceratitis capitata*	GCF_000347755.1	*C. cap*

Table S1: **Input data for the de novo gene annotation in the *Drosophila* clade.** Genomes, proteomes, and annotations were downloaded from the 2016_03 FlyBase release. * = outgroup. Input data for the three outgroup species was acquired from Ensemble Metazoa (*A. gambiae* and *L. cuprina*) and the I5K project (*C. capitata*).

**Age/Ma**	**De novo (ig.)**	**De novo (it.)**	**Put.**	**Div.**	**Tot.**	**% De novo (a.)**
**0.0**	1295	936	2557	1115	5903	37.8
**3.3**	14	16	285	76	391	7.7
**5.9**	87	38	128	36	289	43.3
**8.2**	29	33	33	41	136	45.6
**11.4**	25	25	254	45	349	14.3
**31.1**	16	4	50	34	104	19.2
**33.9**	2	2	11	26	41	9.8
**37.2**	3	7	37	23	70	14.3
**42.3**	8	24	112	21	165	19.4

Table S2: **Number of orphan genes at each phylostratigraphic age.** Number of orphans of each age category shown divided into apparent emergence mechanism. De novo (ig.) = intergenic de novo; De novo (it.); intronic de novo; Put. = putative de novo; Div. = divergent orphans; Tot. = all orphan genes; % De novo (a.) = percentage of orphans found to be de novo (both intergenic and intronic).

	Ka	Ks	(Ka/Ks)	α	p-value	d	f	b
**De novo**	0.044	0.057	0.769	0.192	0.54	0.316	0.612	0.064
**Old**	0.016	0.073	0.216	0.415	<0.001	0.869	0.126	0.005
**Intergenic ORF**	0.024	0.026	0.917	0.064	0.408	0.125	0.859	0.016

Table S3: **Integrative McDonald-Kreitman test for single-exon *D. melanogaster* de novo genes**. A subset of 27 single-exon de novo genes are compared to 106 conserved genes and 747 intergenic ORFs. b = fraction of weakly deleterious sites; d = fraction of strongly deleterious sites; f = neutral fraction.

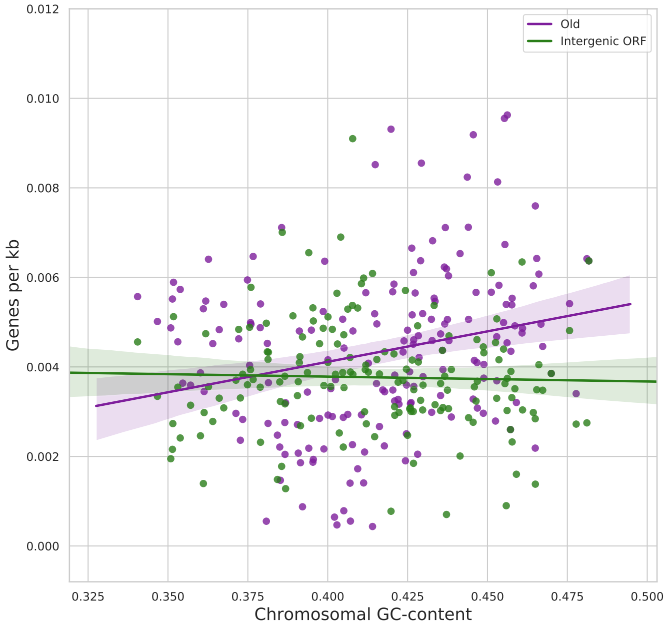

Figure S1: **Gene density correlates with chromosomal GC-content, while density of intergenic ORFs does not**. Subsets of old genes (n=6851) and intergenic ORFs (n=6763) were analysed as for orphan genes (Fig. 2b). Absolute number of genes per kb (y-axis) is in this case arbitrary, but correlation with GC-content of chromosome arms across the twelve *Drosophila* genomes indicates that gene density is positively correlated with chromosome arm GC-content. Old genes: r=0.19, p=0.02; intergenic ORFs: r=-0.01, p=0.90.

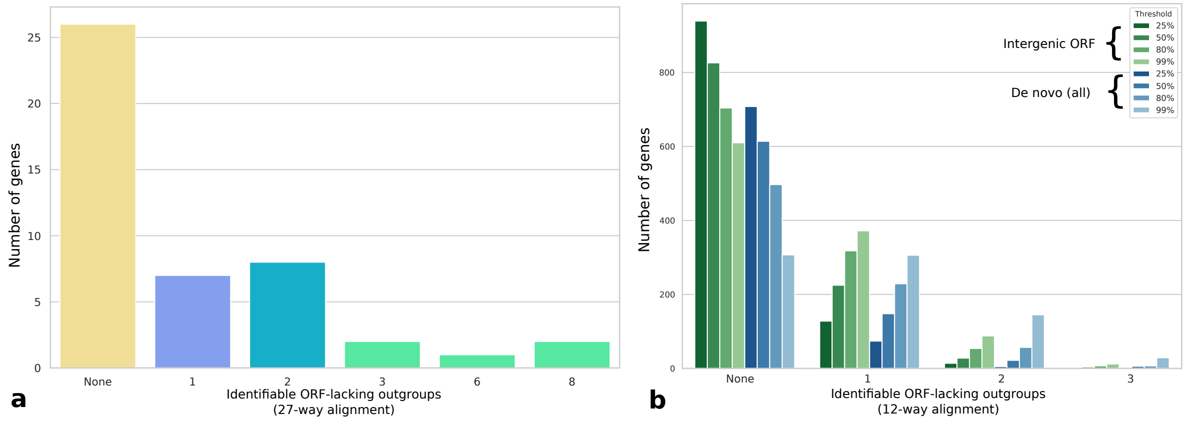

Figure S2: **Identifiable ORF-lacking syntenic outgroups for *Drosophila* de novo genes**. **(a)** Using a 27-way alignment of the *Drosophila* clade (*D. melanogaster* de novo genes only). Analysis was limited to single-exon de novo genes in order to avoid ambiguity over conservation of splice sites in outgroup genomes. Outgroups to a given *D. melanogaster* de novo gene were denoted ORF-harbouring if they contained an ORF overlapping with more than 50% of the focal ORF. ORF status was only inferred in species with intact syntenic regions. **(b)** Using a twelve-way alignment of the *Drosophila* clade (all single exon de novo genes). Analysis of syntenic genomic regions was extended to examine de novo genes across the clade, using a shallower alignment of the twelve *Drosophila* species initially studied. Applying the same criteria to the 771 single exon genes for which an aligned region could be retrieved, we identify 172/771 (22.3%) de novo genes with at least one ORF-lacking outgroup.

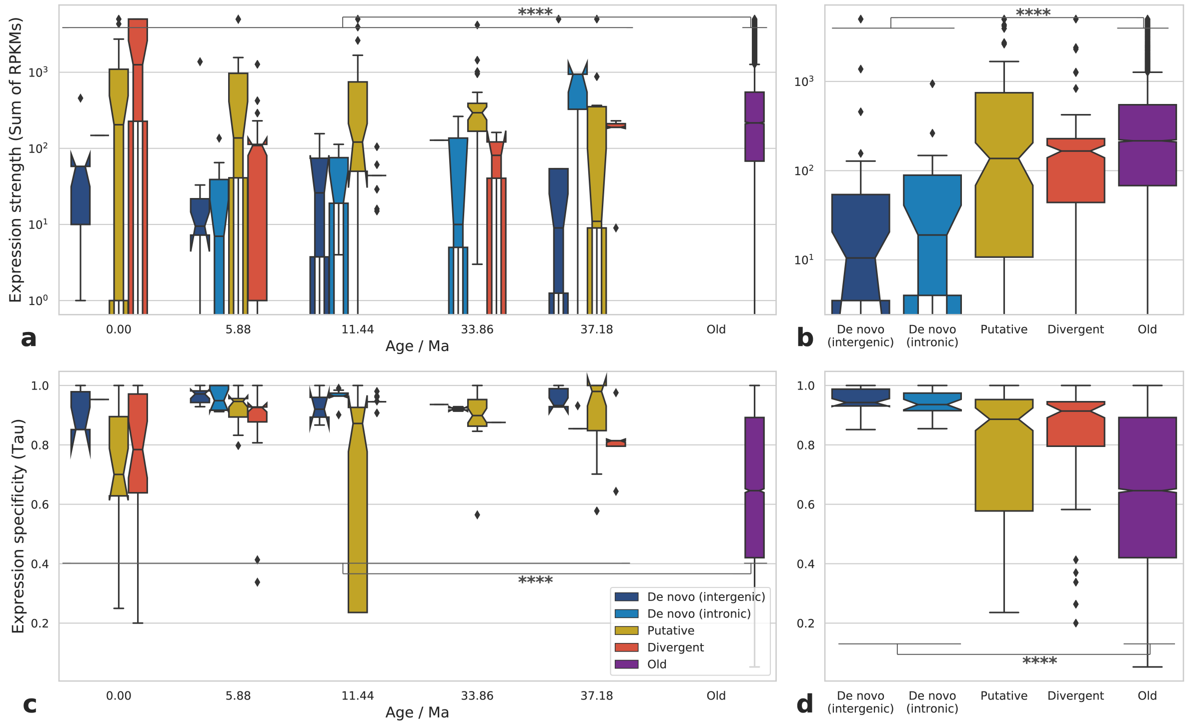

Figure S3: **Analysis of modENCODE RNA-Seq data: strength and specificity of orphan genes in *D. melanogaster*.** **(a)** Expression strength (sum of RPMK values across 29 samples) is shown by gene age: orphans have weaker median expression strength than older genes, with no clear trend visible over the timescale studied. **(b)** Examining expression strength across all ages indicates that de novo genes are more weakly expressed than conserved genes, while divergent orphans show more comparable expression strength to conserved genes. **(c)** Expression specificity (Tau), calculated on log-transformed RPKM data, with a score of 1.0 indicating expression in only one tissue. **(d)** Expression specificity shown by orphan class; all categories of orphan gene show highly biased expression in comparison to that of old genes. Stars indicate significance of Mann-Whitney U test; panels a and c: all orphans vs old genes; panels b and d: de novo (intergenic + intronic) vs old genes.

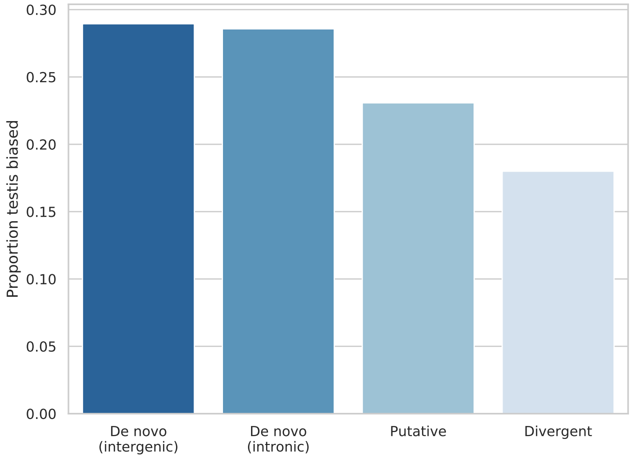

Figure S4: **Proportion of testis biased *D. melanogaster* orphans of each emergence category**. We looked for evidence of testis biased transcription using 29 modENCODE RNA-Seq samples, defining testis biased genes as those with 50% or more of total RPKM confined to testis tissue samples. In total we find that 58/246 (23.6%) orphans have testis biased expression, including 8/66 (12%) de novo genes.

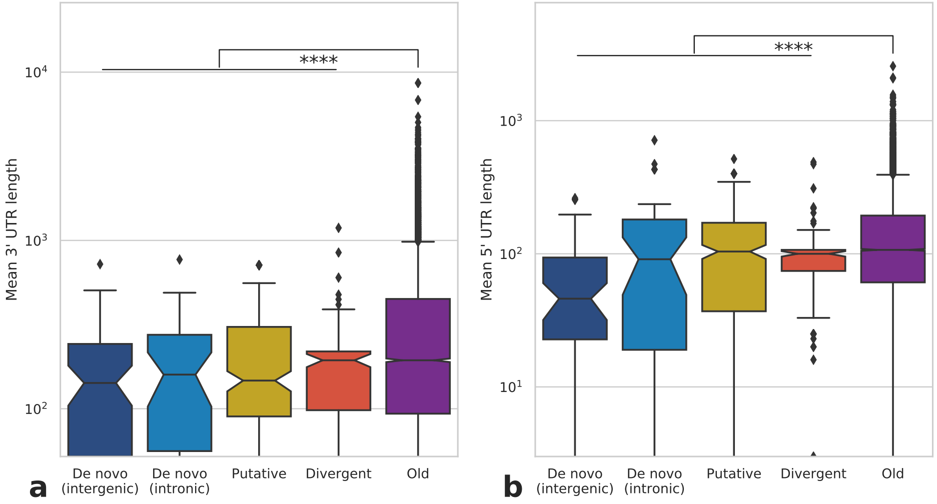

Figure S5: **UTR length of *D. melanogaster* orphans**. **(a)** 3' and **(b)** 5' UTRs are found to be significantly shorter in orphan genes than in conserved genes (log-scale on y-axis). Divergent orphans - which overlap with an outgroup CDS - are likely to be found on more conserved transcripts, reflected in the higher structural maturity of their UTRs when compared to de novo and putative orphans. Stars indicate significance of Mann-Whitney U test for orphan vs old genes.

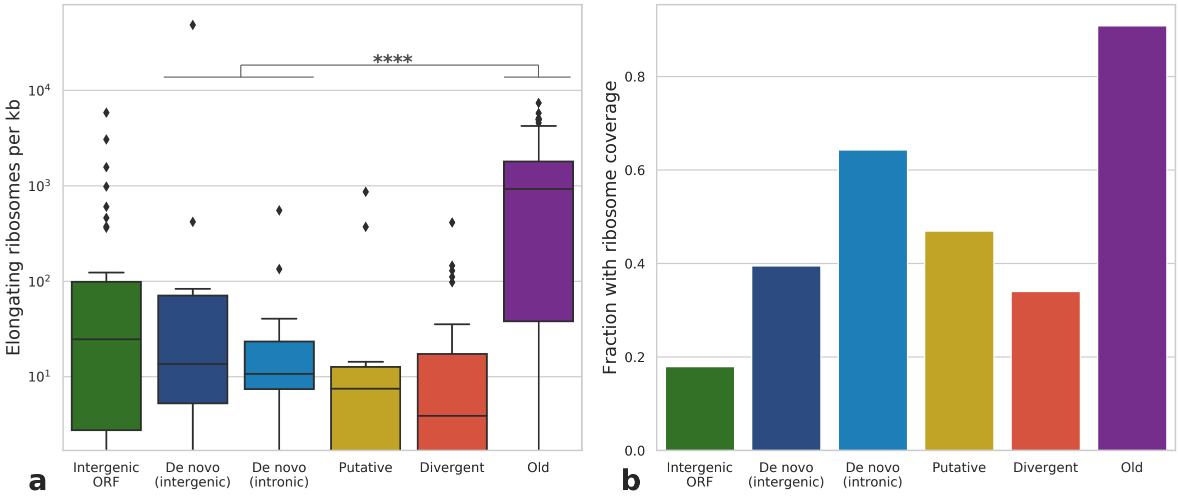

Figure S6: **Analysis of GWIPS ribosome profiling data**. **a**) Ribosome profiling support for sequences in *D. melanogaster*. Density of reads (across three datasets) for elongating ribosomes are shown for each sequence class (calculated as reads per kilobase), illustrating that old genes have significantly higher density of bound ribosomes relative to de novo genes, when they appear in Ribo-Seq datasets. The distribution of densities for de novo genes is comparable to that of intergenic ORFs, with the difference between these categories being statistically indistinguishable. Stars indicate significance of Mann-Whitney U test for de novo (all) vs old genes. **b**) Fraction of sequences with non-zero coverage of elongating ribosomes, including those sequences not found with accompanying RNA-Seq; de novo genes more likely to be bound by a ribosome than intergenic ORFs, but less likely than older genes.

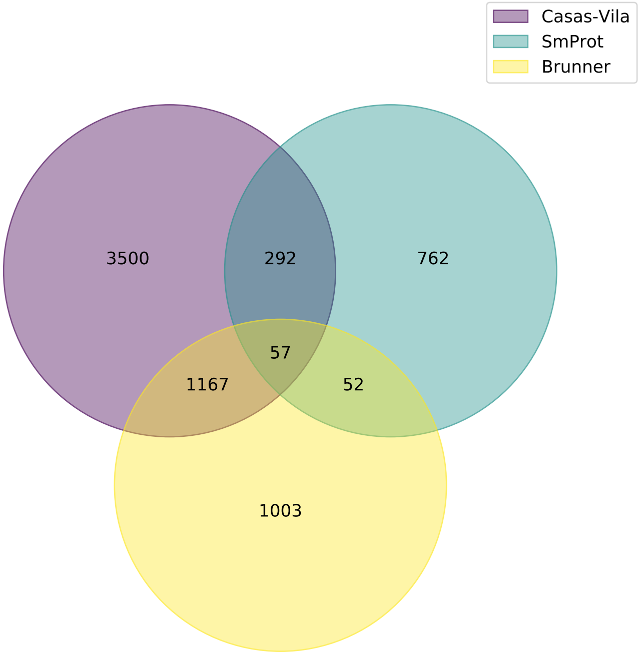

Figure S7: **Additional sources of *D. melanogaster* translational evidence aggregated in this study.** *D. melanogaster* protein-coding genes were checked for their presence in three datasets: Casas-Vila; proteins identified in the developmental proteome by MS (Casas-Vila *et al.* 2017). Brunner; proteins identified in the whole proteome by MS (Brunner *et al.* 2007). SmProt; proteins found in the SmProt database of short proteins with translational evidence from MS, Ribo-Seq and literature sources (Hao *et al.* 2018). Venn diagram shows the intersection of the 6833 unique *D. melanogaster* FlyBase gene identifiers from the three datasets.

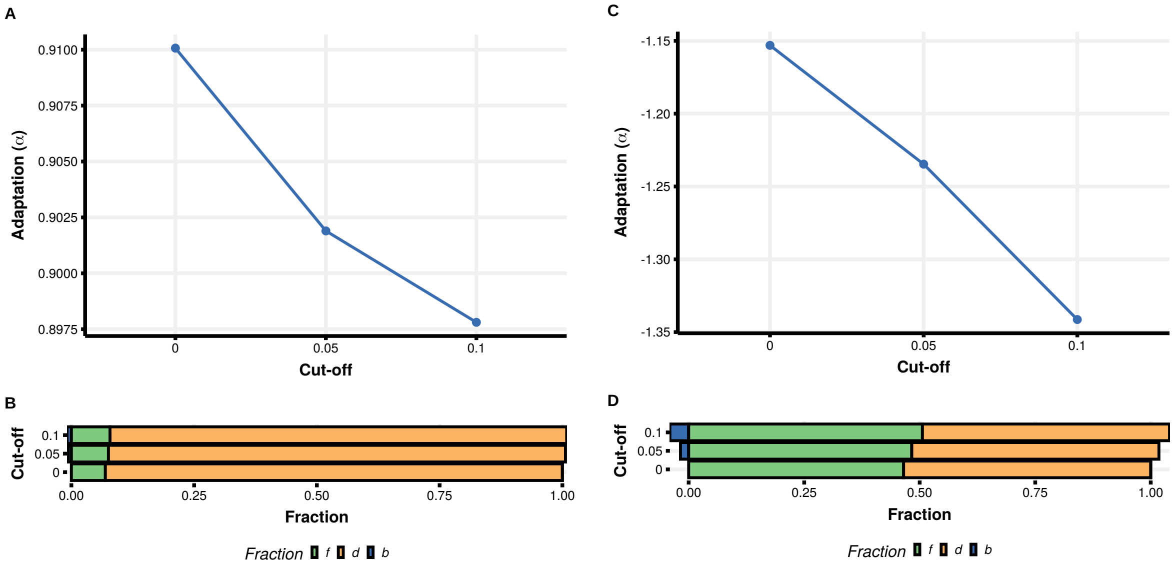

Figure S8: **Integrative McDonald-Kreitman test (iMKT) results for *D. melanogaster* single exon genes.** **(a)** Estimation of α at three confidence thresholds for 27 single-exon de novo genes in *D. melanogaster*. **(b)** Fractions of sites evolving under different selective regimes for de novo genes. Most sites appear to be evolving under a neutral regime. **(c)** Estimation of α at three confidence thresholds for 106 randomly sampled conserved genes. **(d)** Site fractions for conserved genes indicate a higher fraction of deleterious sites: b = weakly deleterious sites; d = strongly deleterious sites; f = neutral fraction.

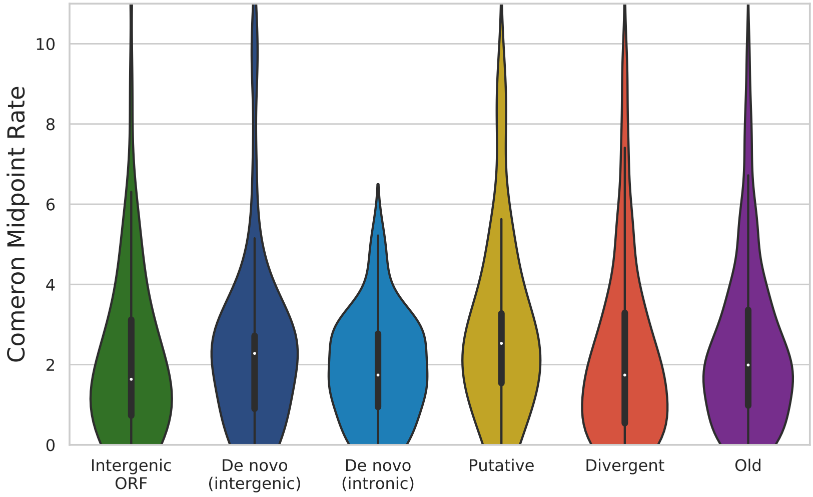

Figure S9: **Recombination rate at *D. melanogaster* gene loci.** Comeron Midpoint Rate describes experimentally determined recombination rates taken for the midpoints of *D. melanogaster* genome coordinates (Comeron *et al.* 2012). De novo (intergenic) vs intergenic ORF: Cohen's d=0.24, Mann-Whitney U p-value=0.44; de novo (intergenic) vs old: Cohen's d= 0.29, Mann-Whitney U p-value=0.16.

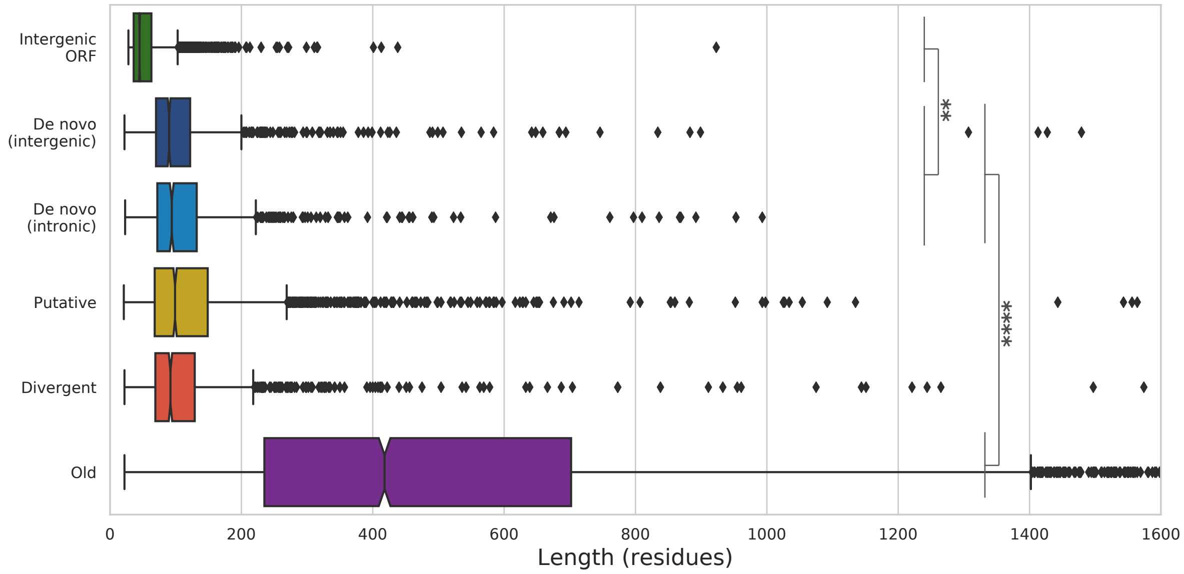

Figure S10: **Length distributions for orphan genes compared to intergenic ORFs and old genes.** All classes of orphan gene have sequence lengths intermediate between random intergenic ORFs (median 47 amino acids) and conserved genes (median 418 amino acids). The median length of all de novo genes across the clade is 81 amino acids. Stars indicate significance of Mann-Whitney *U* test (combined set of de novo genes vs intergenic ORFs or old genes).

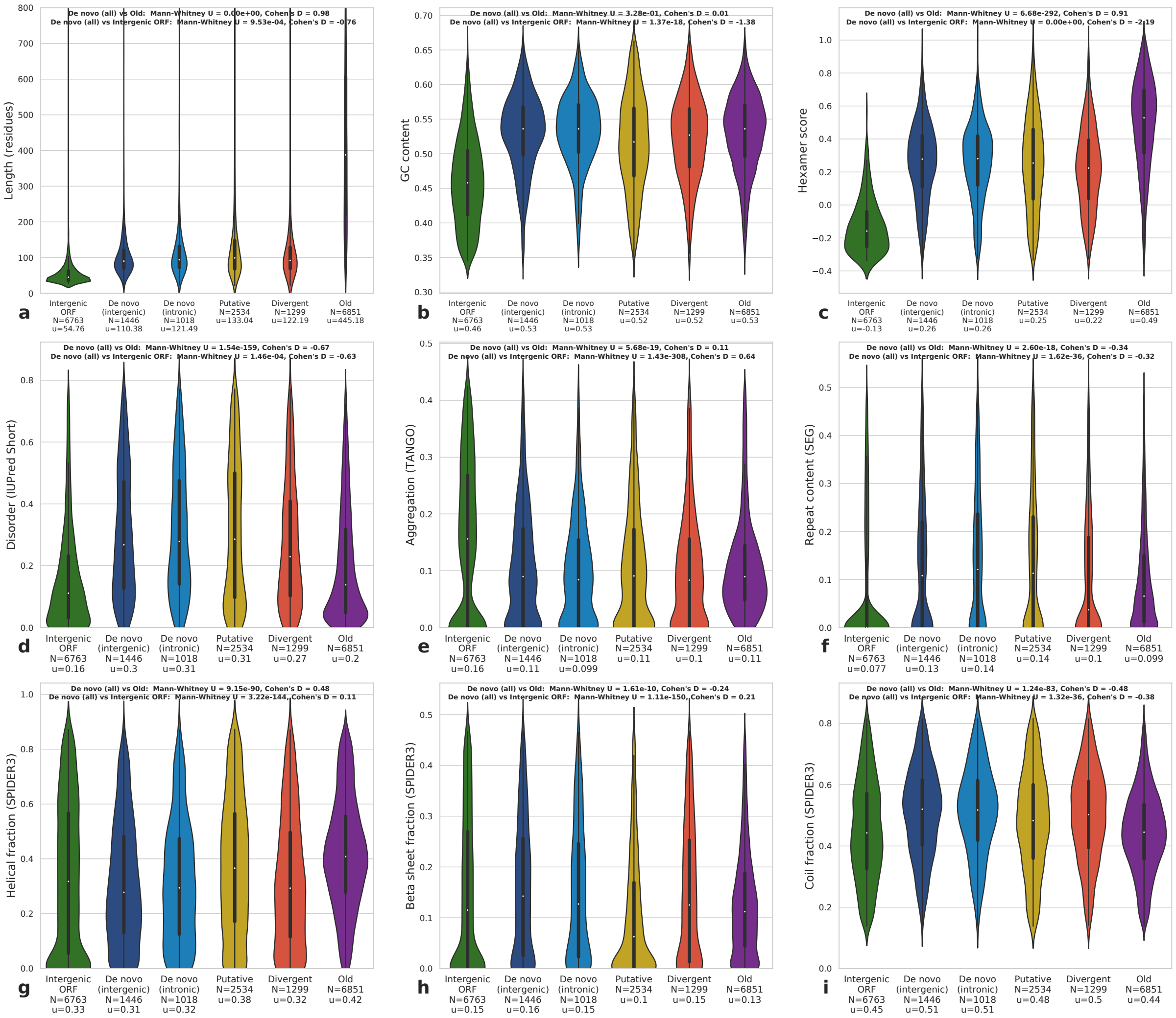

Figure S11: **Sequence properties of de novo proteins in the *Drosophila* clade.** The nucleotide and protein sequence properties of all orphan classes are shown in comparison to sets of randomly chosen old genes and intergenic ORFs. One orphan gene was selected per COG to avoid bias due to phylogenetic distribution. **(a)** Orphan genes are significantly shorter than conserved proteins, but longer than randomly occurring intergenic ORFs. **(b,c)** Nucleotide sequence properties: the GC-content of orphan gene classes is similar to old genes, while for intergenic ORFs it is significantly lower. Hexamer scores for orphan classes are intermediate to intergenic ORFs and older genes. **(d,e)** Protein disorder is elevated in de novo genes and other orphans compared to both intergenic ORFs and older proteins. Aggregation propensity is similar for orphans and older genes, while intergenic ORFs show raised propensity. **(f)** Repeat content at the amino acid level is slightly elevated in de novo and putative classes. **(g,h,i)** Secondary structure propensity; all sequence classes appear to contain abundant helical regions, with old proteins showing a slight elevation relative to orphans. In all cases, significance (Mann-Whitney U) and effect size (Cohen's d) for the difference between the distribution of de novo genes (intergenic and intronic combined) with that of intergenic ORFs and old genes are described at the top of the plot.

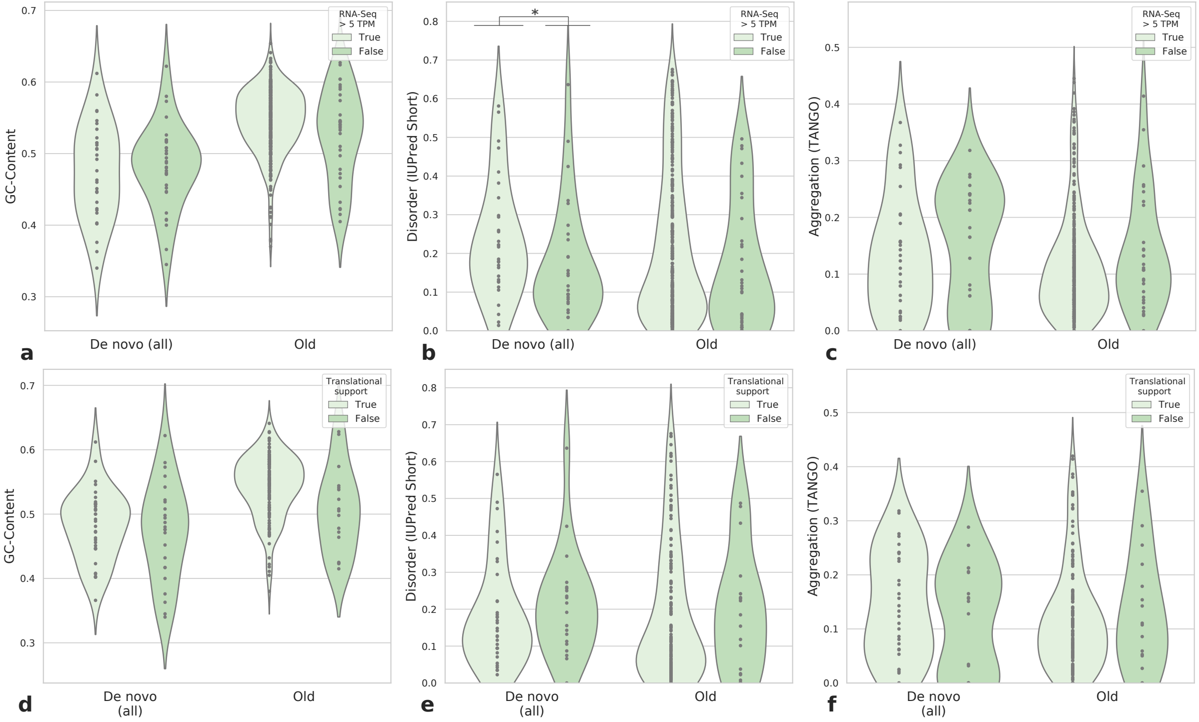

Figure S12: **Comparison of sequence properties of *D. melanogaster* genes when split by strength of RNA-Seq and translational evidence**. **(a-c)** RNA-Seq: GC-content, disorder and aggregation propensity for de novo and old genes, split by robust expression. We set a stringent threshold for robust expression as presence in 711 or more samples (5% of the 14,423 SRA samples), at a level of 5 TPM or greater. Genes with high RNA-Seq evidence; n=34. Genes with low RNA-Seq evidence; n=32. **(d-f)** Translational support: genes split by presence or absence of translational support in MS data and literature sources and Ribo-Seq data. Genes with translational evidence; n=37. Genes without translational evidence; n=24. Stars indicate significance based on the Mann-Whitney U test; only significant comparison shown in panel b.

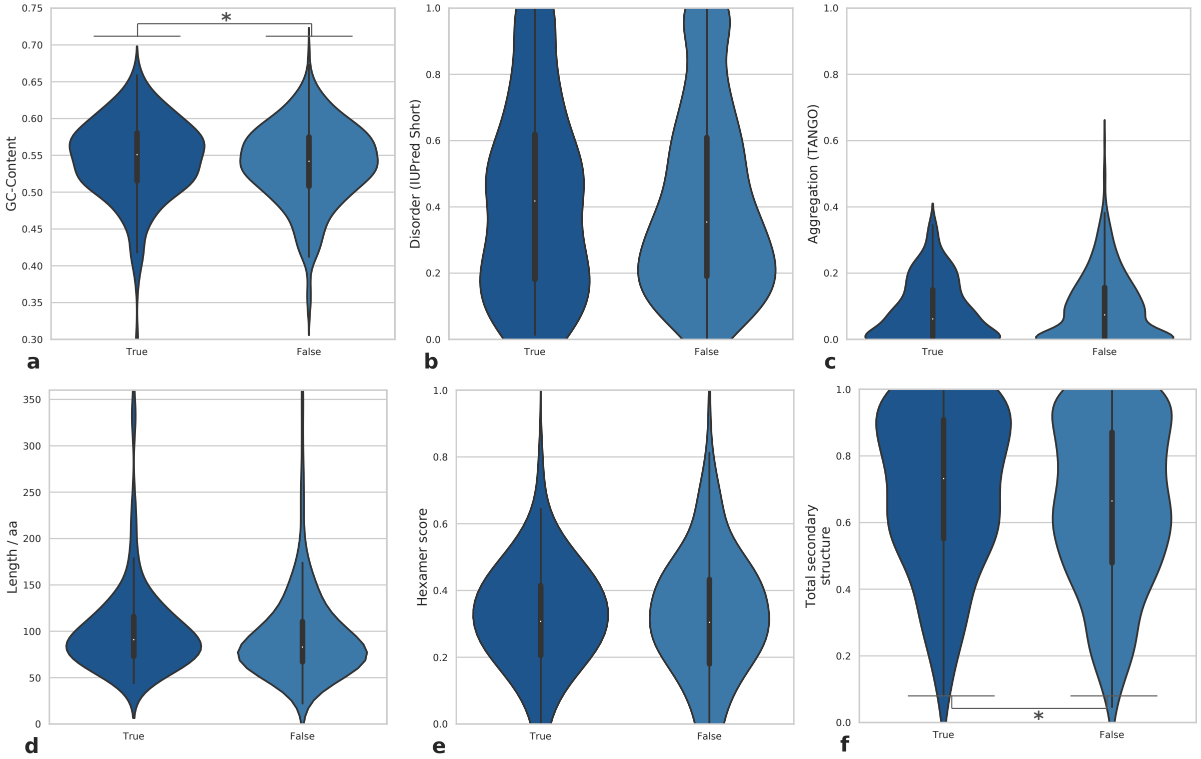

Figure S13: **Comparison of sequence properties of *Drosophila* de novo genes with (n=172) and without (n=599) validation by one or more ORF-lacking outgroup**. We split the subset of de novo genes for which we could examine ORF conservation across a 12-way genome alignment of the *Drosophila* clade into those for which ORF emergence is unambiguous ('True', with one or more ORF-lacking outgroups) and less clear ('False'). We find that properties of these subsets are robust to partitioning based on this criterion. For each sequence property we also computed Cohen's d effect size for the difference between distributions, and Mann-Whitney U for the significance of any difference (p-values):
(a) GC-content: Cohen's d=0.14, p=0.02.
(b) Disorder: Cohen's d=0.05, p=0.17.
(c) Aggregation: Cohen's d=-0.04, p=0.45.
(d) Length: Cohen's d=-0.03, p=0.18.
(e) Hexamer score: Cohen's d=-0.03, p=0.46.
(f) Total secondary structure: Cohen's d=0.17, p=0.04.

Protein	Gene	Mech.	Age (My)	Chrom.	L (nt)	Hexamer	d_N/d_S	Disord.	Aggn.	RMPK-sum	Tau
FBpp0401593	FBgn0265538	denovo-intergen	0	2R	93	-0.12	0.41	0.15	0.52	457	1
FBpp0401603	FBgn0262857	denovo-intergen	0	2L	215	-	-	-	-	1	1
FBpp0311647	FBgn0266845	denovo-intergen	0	2L	81	0.26	99	0.91	0	7	0.99
FBpp0312119	FBgn0267254	denovo-intergen	5.88	2L	138	-0.4	0.41	0.19	0.32	2	1
FBpp0310944	FBgn0266534	denovo-intergen	5.88	3R	1041	-0.12	0.47	0.49	0.07	5	0.86
FBpp0311135	FBgn0266591	denovo-intergen	5.88	3L	357	-0.06	0.36	0.03	0.26	10	1
FBpp0310416	FBgn0266261	denovo-intergen	5.88	3R	489	-0.26	0.2	0.08	0.08	1377	1
FBpp0297200	FBgn0262813	denovo-intergen	11.44	3L	246	-0.15	0.14	0.18	0.06	0	-
FBpp0302845	FBgn0263841	denovo-intergen	33.86	3L	225	0.15	2.62	0.04	0.12	128	0.98
FBpp0310829	FBgn0266456	denovo-intergen	37.18	3L	279	0.39	0.48	0.02	0.14	11414	1
FBpp0300322	FBgn0052106	denovo-intergen	37.18	3L	4242	-0.26	-	0.57	0.02	54	0.95
FBpp0075890	FBgn0053272	denovo-intergen	37.18	3L	267	0.22	-	0.09	0.23	8	0.98
FBpp0075881	FBgn0053271	denovo-intergen	37.18	3L	267	0.29	-	0.09	0.24	0	-
FBpp0075880	FBgn0053270	denovo-intergen	37.18	3L	267	0.19	-	0.09	0.24	3	0.98
FBpp0075825	FBgn0053500	denovo-intergen	37.18	3L	267	0.15	-	0.09	0.24	16	0.99
FBpp0297197	FBgn0262811	denovo-intergen	37.18	X	216	0.08	0.29	0.29	0.31	2	1
FBpp0297277	FBgn0262823	denovo-intergen	37.18	3R	267	0.12	0.5	0.07	0.28	9	0.71
FBpp0292442	FBgn0036586	denovo-intergen	37.18	3L	459	0.2	-	0.05	0.23	0	-
FBpp0312443	FBgn0266977	denovo-intergen	37.18	3L	288	0.09	0.74	0.14	0.05	47	0.97
FBpp0085932	FBgn0050114	denovo-intergen	37.18	2R	348	0.13	0.24	0.05	0.06	2	0.96
FBpp0312040	FBgn0267163	denovo-intron	5.88	X	366	0.07	0.85	0.18	0.1	136	0.99
FBpp0311425	FBgn0266745	denovo-intron	5.88	3R	255	-0.2	-	0.19	0.16	7	0.91
FBpp0309529	FBgn0265762	denovo-intron	5.88	2R	249	-0.25	0.43	0.33	0.06	0	-
FBpp0297192	FBgn0262818	denovo-intron	5.88	2R	177	0.09	99	0.38	0	0	-
FBpp0310029	FBgn0266050	denovo-intron	5.88	X	360	-0.29	0.28	0.03	0.27	0	-
FBpp0309190	FBgn0265576	denovo-intron	5.88	2R	444	0.06	1.14	0.78	0	65	0.95
FBpp0309376	FBgn0265608	denovo-intron	5.88	X	270	0.32	0.32	0.1	0.18	0	-
FBpp0291587	FBgn0261501	denovo-intron	5.88	2R	219	0.11	0.26	0.12	0.24	1	1
FBpp0309112	FBgn0265535	denovo-intron	5.88	X	840	0.45	-	0.22	0.03	22	0.97
FBpp0082695	FBgn0051088	denovo-intron	11.44	3R	402	-0.18	-	0.17	0.19	113	0.94
FBpp0306786	FBgn0262890	denovo-intron	11.44	3L	201	-0.06	-	0.22	0	19	0.87
FBpp0301044	FBgn0029707	denovo-intron	11.44	X	285	0.4	-	0.47	0.09	4	0.89
FBpp0074391	FBgn0030936	denovo-intron	33.86	X	999	0.32	0.83	0.13	0.13	263	0.98
FBpp0293417	FBgn0262541	denovo-intron	33.86	3R	441	-0.2	-	0.13	0.11	10	0.86
FBpp0075656	FBgn0036311	denovo-intron	37.18	3L	816	0.23	0.47	0.41	0.02	942	0.97
FBpp0111333	FBgn0085254	denovo-intron	37.18	2R	315	0.18	0.77	0.34	0.17	0	-
FBpp0070140	FBgn0025616	denovo-intron	37.18	X	1482	0.23	0.48	0.16	0.02	121	0.98

Table S4: **De novo genes found in *D. melanogaster* with translational support.**

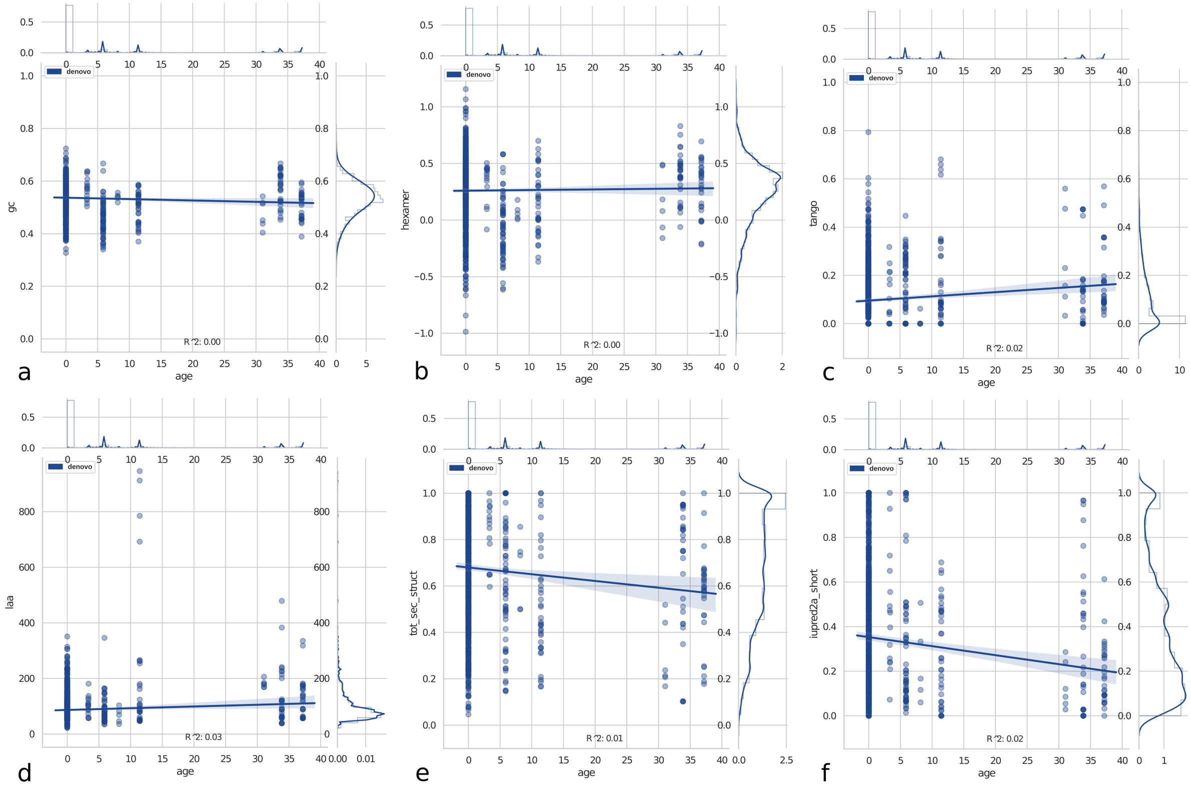

Figure S14: **Correlations of sequence properties with age for de novo genes across the *Drosophila* clade.** In all plots, inferred gene age (in Ma) is shown on the x-axis. R^2 for linear regression illustrated in each panel. a) GC-content; b) Hexamer score; c) Aggregation propensity; d) Length (amino acids); e) Total secondary structure (alpha helix + beta sheet); f) Disorder.

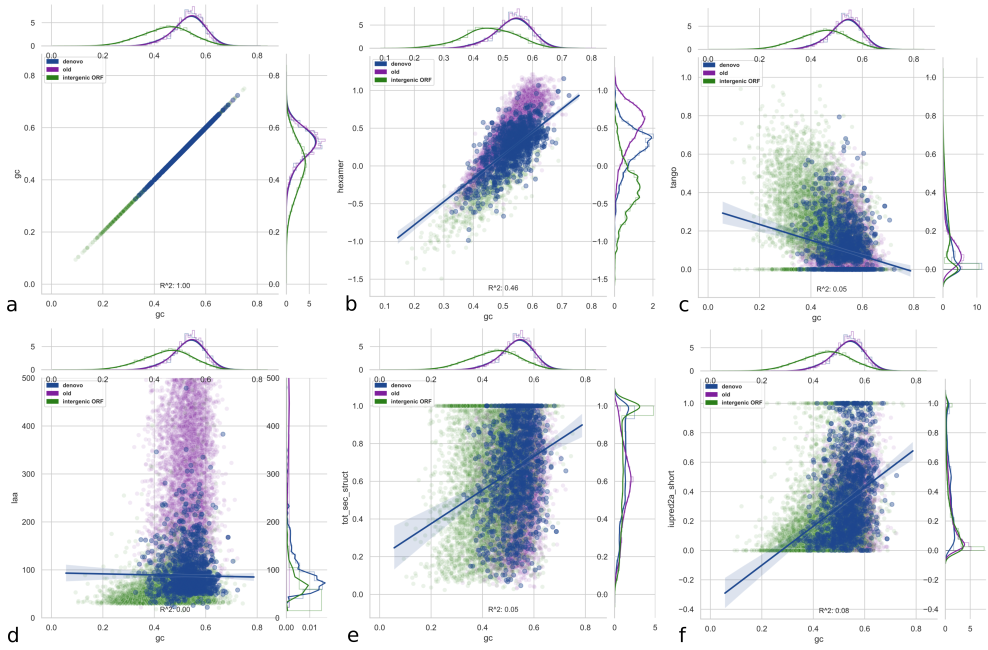

Figure S15: **Correlations of sequence properties with GC-content for de novo genes across the *Drosophila* clade.** In all plots, fractional GC-content is shown on the x-axis. R^2 for linear regression of de novo gene set illustrated in each panel. a) GC-content; b) Hexamer score; c) Aggregation propensity; d) Length (amino acids); e) Total secondary structure (alpha helix + beta sheet); f) Disorder.

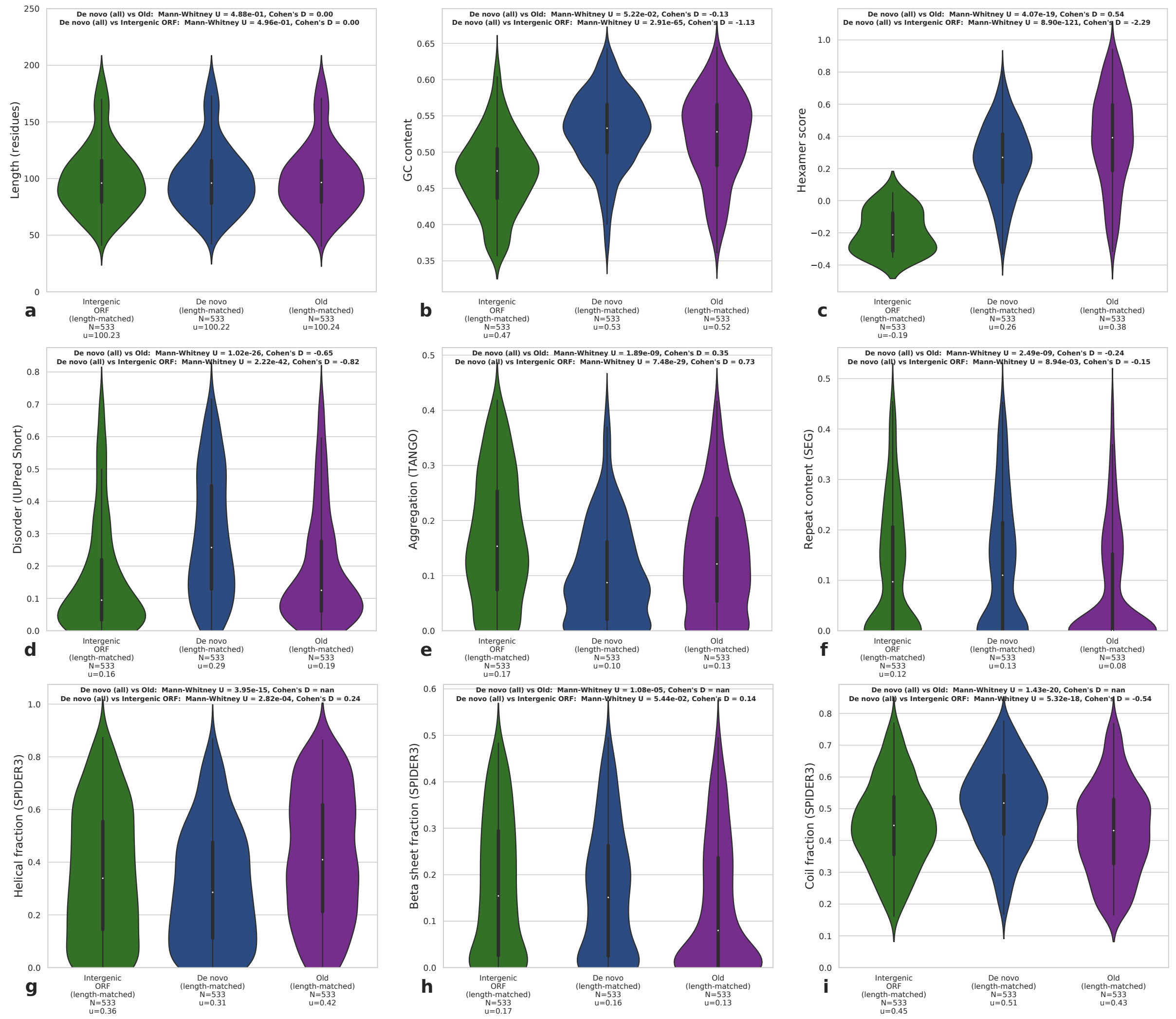

Figure S16: **Sequence property analysis of length-matched subsets of de novo genes, intergenic ORFs and old genes.** Sequences were first binned into subsets of increasing length (at intervals of 10 amino acids). For each de novo gene, one sequence each was then selected at random from the subset of intergenic ORFs and old genes assigned to the same length bin to create three matched sets of sequences (N=533 for each class); see length distributions in panel a. In all cases, significance (Mann-Whitney U) and effect size (Cohen's d) for the difference between the distribution of de novo genes (intergenic and intronic combined) with that of intergenic ORFs and old genes are described at the top of the plot.

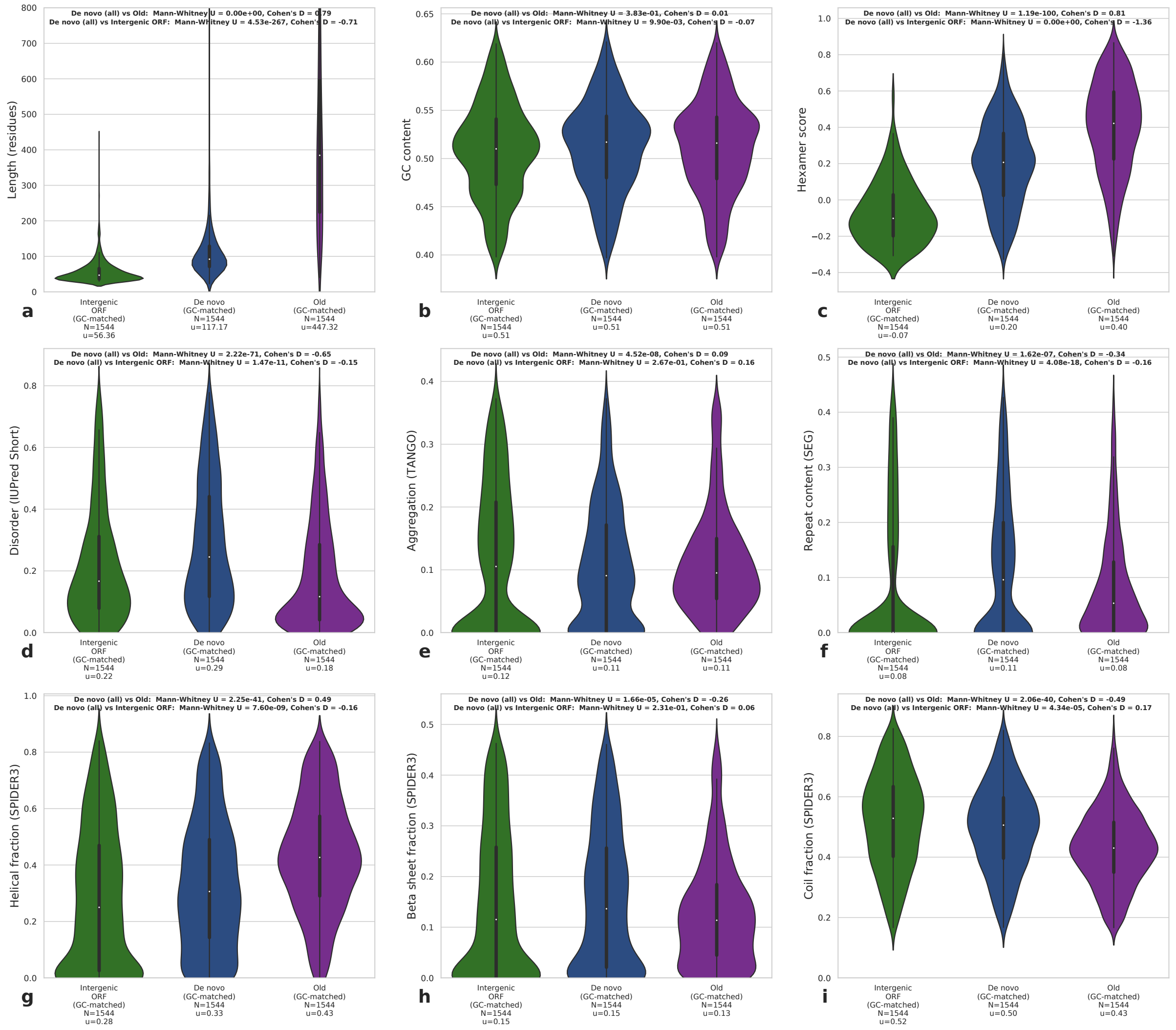

Figure S17: **Sequence property analysis of GC-content-matched subsets of de novo genes, intergenic ORFs and old genes.** Sequences were first binned into subsets of increasing GC-content (at intervals of 5% GC-content). For each de novo gene, one sequence each was then selected at random from the subset of intergenic ORFs and old genes assigned to the same GC-content bin to create three matched sets of sequences (n=1544 for each class); see GC-content distributions in panel b. In all cases, significance (Mann-Whitney U) and effect size (Cohen's d) for the difference between the distribution of de novo genes (intergenic and intronic combined) with that of intergenic ORFs and old genes are described at the top of the plot.
